# Use of 3-[^18^F]fluoropropanesulfonyl chloride as a prosthetic agent for the radiolabelling of amines: Investigation of precursor molecules, labelling conditions and enzymatic stability of the corresponding sulfonamides

**DOI:** 10.3762/bjoc.9.115

**Published:** 2013-05-27

**Authors:** Reik Löser, Steffen Fischer, Achim Hiller, Martin Köckerling, Uta Funke, Aurélie Maisonial, Peter Brust, Jörg Steinbach

**Affiliations:** 1 Institute of Radiopharmaceutical Cancer Research (formerly Institute of Radiopharmacy), Helmholtz-Zentrum Dresden-Rossendorf (HZDR), Bautzner Landstraße 400, 01328 Dresden, Germany; 2 Department of Chemistry and Food Chemistry, Technical University of Dresden, Bergstraße 66c, 01062 Dresden, Germany; 3 Institute of Radiopharmaceutical Cancer Research, HZDR Research Site Leipzig, Permoserstraße 15, 04318 Leipzig, Germany; 4 Institute of Chemistry, University of Rostock, Inorganic Solid-State Chemistry Group, Albert-Einstein-Straße 3a, 18059 Rostock, Germany

**Keywords:** fluorine-18, hydrolytic metabolism, prosthetic groups, radiochemistry, sulfonamides

## Abstract

3-[^18^F]Fluoropropanesulfonyl chloride, a recently proposed prosthetic agent for fluorine-18 labelling, was prepared in a two-step radiosynthesis via 3-[^18^F]fluoropropyl thiocyanate as an intermediate. Two benzenesulfonate-based radiolabelling precursors were prepared by various routes. Comparing the reactivities of 3-thiocyanatopropyl nosylate and the corresponding tosylate towards [^18^F]fluoride the former proved to be superior accounting for labelling yields of up to 85%. Conditions for a reliable transformation of 3-[^18^F]fluoropropyl thiocyanate to the corresponding sulfonyl chloride with the potential for automation have been identified. The reaction of 3-[^18^F]fluoropropanesulfonyl chloride with eight different aliphatic and aromatic amines was investigated and the identity of the resulting ^18^F-labelled sulfonamides was confirmed chromatographically by comparison with their nonradioactive counterparts. Even for weakly nucleophilic amines such as 4-nitroaniline the desired radiolabelled sulfonamides were accessible in satisfactory yields owing to systematic variation of the reaction conditions. With respect to the application of the ^18^F-fluoropropansulfonyl group to the labelling of compounds relevant as imaging agents for positron emission tomography (PET), the stability of *N*-(4-fluorophenyl)-3-fluoropropanesulfonamide against degradation catalysed by carboxylesterase was investigated and compared to that of the analogous fluoroacetamide.

## Introduction

The importance of molecular imaging, i.e., the characterisation and measurement of biological processes in living organisms at the molecular level using remote imaging detectors, for both research and diagnostic purposes has considerably increased over the recent years. The success of this interdisciplinary field depends substantially on the development of molecular probes equipped with appropriate reporter groups [1].

Among the different imaging modalities, positron emission tomography (PET) stands out with regards to sensitivity and quantitative image evaluation. PET is based on the application of molecules labelled with a positron-emitting radionuclide, which are termed radiotracers. Although such radionuclides are known for many elements, fluorine-18 can be considered as the most suitable one for PET due to its intermediate half-life of 109.8 min, its high content of β^+^-conversion (97%) and its rather low positron energy maximum of 640 keV [2].

From a chemical point of view, the introduction of fluorine-18 into molecules that are able to address biomolecular targets in vivo, requires a carefully developed methodology as the carbon–fluorine bond is rather difficult to tie [3–4]. Furthermore, as fluorine appears less frequently in biologically active compounds, molecules that show the potential to interact with certain imaging targets have to be modified with fluorine. For this purpose, generic groups that allow both derivatisation with fluorine as well as convenient introduction of radiofluorine are often used. These groups are referred to as prosthetic groups in preparative radiochemistry. For labelling with fluorine-18, a variety of prosthetic groups were suggested and developed [5–6]. Their careful individual selection is critical for radiotracer development as they often exert great influence on target binding and/or stability in vivo. This is particularly valid when PET imaging probes based on small molecules are considered.

**Figure 1:**
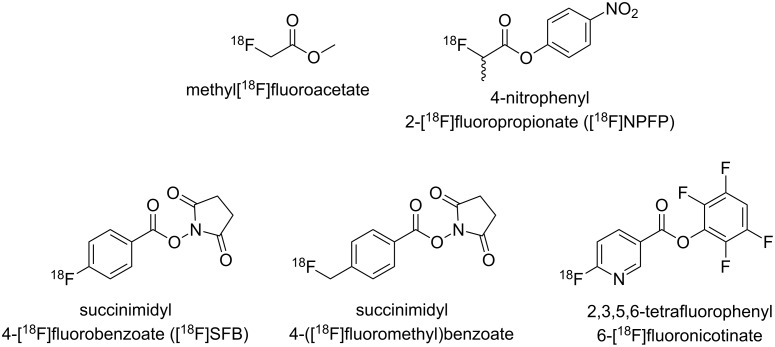
Selection of prosthetic agents for ^18^F-labelling via acylation.

Labelling based on the formation of carboxylic amides is an approach that allows convenient introduction of fluorine-18 (Figure 1) [7–8], which applies especially to [^18^F]fluoroacetamides [9–15]. In several cases, [^18^F]fluoroacetamides were proven to be metabolically unstable due to hydrolytic cleavage [15–17]. As an alternative to acyl-based prosthetic groups the 3-[^18^F]fluoropropanesulfonyl group introduced by Li et al. attracted our interest [18]. Labelling with radiofluorine by sulfonamide formation seems to be intriguing not only because of the inertness against the metabolic cleavage of the label but also because of the polarity it can confer to the resulting tracer molecule. This can be an advantage especially for radiotracers based on small molecules [19]. Therefore, we planned to establish and to optimise the preparation of 3-[^18^F]fluoropropanesulfonyl chloride in our labs and to study its reaction with a panel of aliphatic and aromatic amines of varying reactivity. Particular attention was paid to the synthesis of precursor molecules suitable for radiofluorination and nonradioactive reference compounds, as the information published in [18] is rather preliminary in this regard. Furthermore, we aimed to extend ^18^F-fluoropropanesulfonylation to the labelling of aromatic amines. Additionally, the metabolic stability of 3-fluoropropanesulfonamides was proven and comparatively assessed to that of analogous fluoroacetamides by degradation experiments with carboxylesterase from pig liver. Preliminary results of this study have been published previously as a conference abstract [20].

## Results and Discussion

### Synthesis of precursors and nonradioactive reference compounds

The preparation of ^18^F-labelled sulfonyl chlorides is challenging as the chlorine atom in these electrophilic agents can be exchanged by reaction with fluoride even in the presence of water to form the corresponding sulfonyl fluorides [21]. Hence, the sulfonyl chloride has to be generated by interconversion of a different, less reactive sulfur-containing functional group after radiofluorination. Sulfonyl chlorides can be generated by oxidation with aqueous chlorine from a variety of organosulfur species such as thiols, sulfides, disulfides, thioesters, isothiouronium salts, xanthates and thiocyanates [22]. The latter class of organic sulfur compounds seems to be most advantageous, as organic thiocyanates are easily accessible, sufficiently stable to oxidation, and nonhygroscopic. Li et al. [18] decided to use a propyl spacer between the fluorine-18 atom and the thiocyanate moiety as radiofluorination by nucleophilic substitution proceeds easier at aliphatic than at aromatic electrophilic centres. In addition, the propyl spacer accounts for a balance between the limited size of the prosthetic unit on the one hand and the limited volatility of the radiofluorinated intermediates on the other. Thus, the general approach presented in [18] to generate ^18^F-labelled sulfonyl chlorides seems to be well-conceived and was therefore adopted for our purposes.

**Scheme 1:**
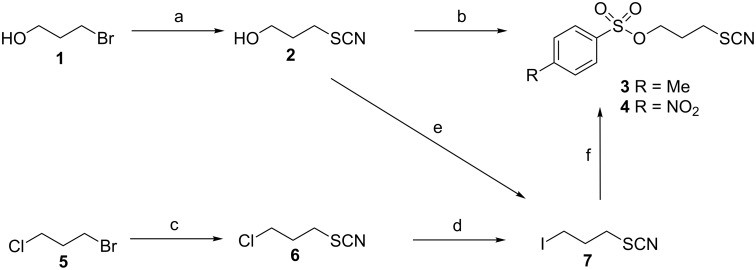
Synthesis of radiofluorination precursors **3** and **4**. Reagents and conditions: (a) KSCN, CH_3_OH, reflux; (b) TsCl, DIPEA, pyridine, CH_2_Cl_2_, rt (for **3**) or NsCl, K(CH_3_)_3_SiO, THF, rt (for **4**); (c) KSCN, CH_3_OH, reflux; (d) NaI, acetone, reflux; (e) Ph_3_P, I_2_, imidazole, THF, rt; (f) silver tosylate (for **3**) or silver nosylate (for **4**), CH_3_CN, rt.

Initially, the route described by Li et al. [18] was followed to synthesise the tosylate precursor **3** (Scheme 1). As the tosylation of the alcohol **2** proceeded in low yields and led to side products that were difficult to remove and impaired the reaction with [^18^F]fluoride, an alternative procedure to afford **3** was envisaged. Esters of sulfonic acids can be also prepared by nucleophilic displacement of carbon-bound halogens with sulfonates, which works best with alkyl iodides and silver salts of sulfonic acids [23]. Therefore, the required 3-iodopropyl thiocyanate (**7**) was synthesised by subjecting the corresponding chloro-derivative **6** to the conditions of a Finkelstein reaction. Unexpectedly, this led to a mixture containing 1,3-diiodopropane and 1,3-dithiocyanatopropane beside **7**, as revealed by ^1^H NMR analysis. From this mixture, the desired product **7** was isolated by distillation in a yield of 24%. Reduction of the amount of sodium iodide from 5 to 1.1 equivalents did not result in a more favourable product distribution. The course of this reaction becomes clear in the light of the pseudohalide concept: the thiocyanate functionality acts as a leaving group towards attack by iodide forming 1,3-diiodopropane. The thereby-released thiocyanate anion reacts with concomitantly formed **7** to give 1,3-dithiocyanatopropane. The reversibility of the iodide/thiocyanate displacement has been reported previously [24] and alkyl thiocyanates can be quantitatively transformed under controlled conditions into the corresponding iodides [25]. The preparation of compound **7** can be also achieved by the transformation of alcohol **2** in an Appel-type reaction, circumventing the problems encountered during the Finkelstein reaction. Conversion of **7** with silver tosylate proceeded smoothly leading to the desired tosylate **3**. In analogy, the nosylate **4** was obtained by reaction of the iodide **7** with silver nosylate, which was prepared according to a published procedure [26]. Alternatively, **4** was obtained by converting alcohol **2** with nosyl chloride. This procedure resulted in lower yields but can be considered as more efficient, as it is shorter by one step.

**Scheme 2:**
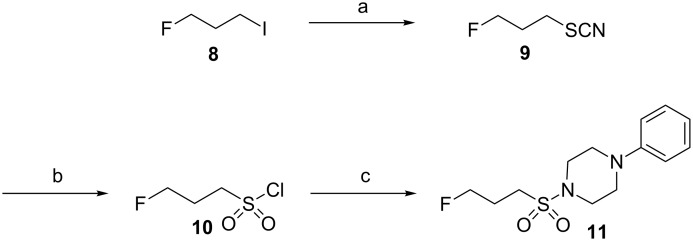
Synthesis of 3-fluoropropanesulfonamide **11** via intermediary 3-fluoropropanesulfonyl chloride (**10**). Reagents and conditions: (a) KSCN, CH_3_OH, reflux; (b) Cl_2_, H_2_O/AcOH, rt; (c) *N*-phenylpiperazine, triethylamine (TEA), CH_2_Cl_2_, reflux.

**Scheme 3:**
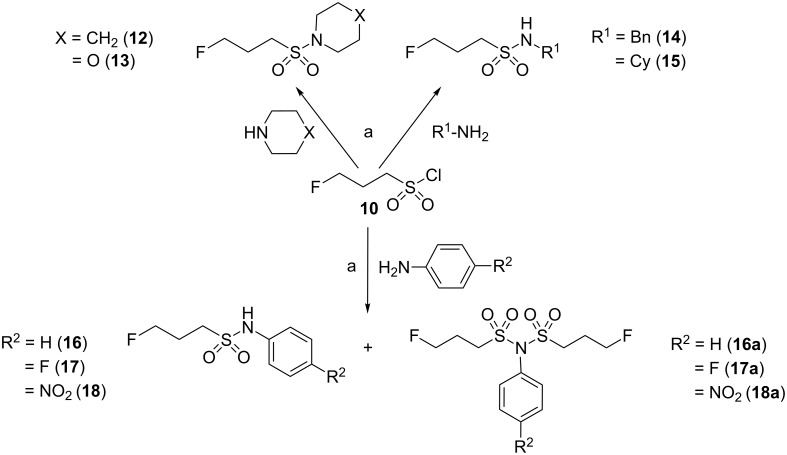
Synthesis of 3-fluoropropanesulfonamides **12**–**18**. Reagents and conditions: (a) triethylamine (TEA), CH_2_Cl_2_, reflux (for **12**–**17**) or pyridine, CH_2_Cl_2_, rt (for **18**).

The synthesis of the ^19^F-based reference compounds started with the conversion of commercially available 1-fluoro-3-iodopropane (**8**) with potassium thiocyanate, analogous to the preparation of **2** (Scheme 2). The key step was the transformation of the thiocyano group of **9** to the chlorosulfonyl group leading to 3-fluoropropanesulfonyl chloride (**10**). This functional group interconversion using aqueous chlorine has been known for a long time [27] but has received little attention in synthetic organic chemistry. Following this approach, Millington et al. were able to obtain the sulfonylating agent **10** by saturating an aqueous suspension of thiocyanate **9** with chlorine gas [28]. In our hands, superior results were achieved when this transformation was carried out in a mixture of chlorine-saturated water and acetic acid as cosolvent for **9**. The desired intermediate was purified by vacuum distillation or transformed as crude product to the final 3-fluoropropanesulfonamides, as shown for compound **11**. Alternatively, **10** was obtained commercially. The reaction of **10** with aliphatic amines proceeded quantitatively and smoothly to the sulfonamides **12**–**15** (Scheme 3). In contrast, its reaction with aniline derivatives required a longer time and led to the formation of side products that were identified as the corresponding *N*,*N*-bissulfonylanilines **16a**–**18a**. The double sulfonylation of aromatic amines under strongly basic conditions was described earlier [29]. This side reaction was most pronounced for the reaction of **10** with 4-nitroaniline, which resulted in the formation of **18a** as the main product. Therefore, **10** was reacted with 4-nitroaniline in the presence of pyridine at room temperature resulting in the incomplete conversion to the mono-sulfonylated product **18**. This compound was obtained in the form of crystals suitable for X-ray diffraction analysis.

**Figure 2:**
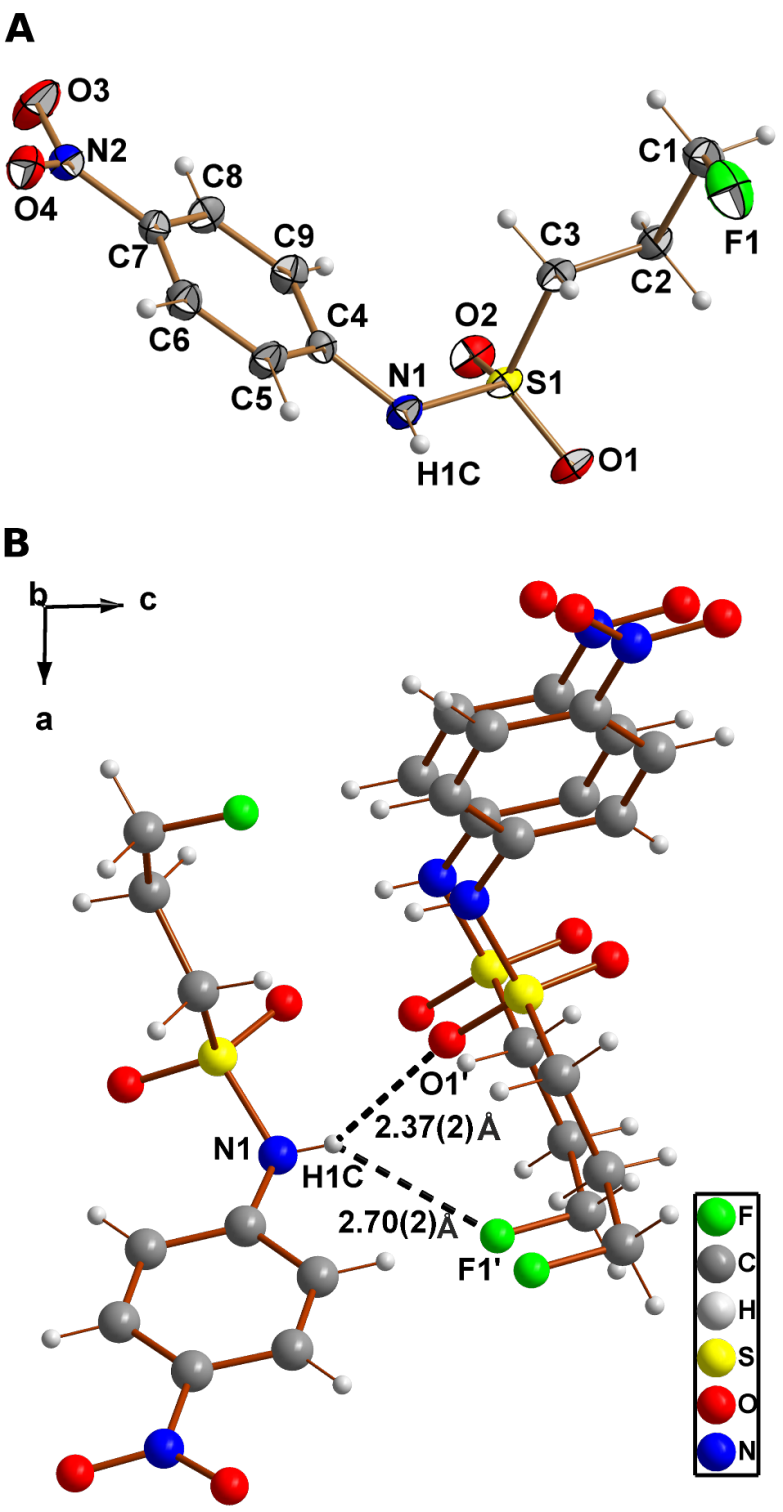
(**A**) View of the molecular structure of sulfonamide **18** with atom labelling scheme. Displacement ellipsoids are drawn at the 50% probability level. (**B**) View of the arrangement of molecules in crystals of sulfonamide **18** showing hydrogen-bond contacts of the amide H-atom H1C.

The molecular structure of **18** is shown in Figure 2A, confirming unambiguously its identity as *N*-(4-nitrophenyl)-3-fluoropropane-1-sulfonamide. Crystal data and structure refinement parameters are collected in Table 1. The length of the S–N bond in compound **18** is with 1.639(1) Å close to the average value of 1.63(2) Å observed for sulfonamides [30]. Whereas the nitrogen atom of carboxylic amides is typically trigonal planar, that of sulfonamides tends to be pyramidalised [31–32]. This phenomenon can be also observed for the molecular structure of **18** in the crystal. This is indicated by the fact that the sum of the three valence angles around the sulfonamide nitrogen (C4–N1–S1, C4–N1–H1C, and S1–N1–H1C) is equal to 348(4)°, which is significantly less than 360° for trigonal planar geometry. Further evidence for pyramidalisation is provided by the out-of-plane angle for S1 (sulfonamide sulfur atom) with respect to the plane defined by the atoms N1, C4 and H1C, which is 34(2)° compared to zero for the trigonal planar shape. The orientation of the N1 lone electron pair is antiperiplanar to the S1–C3 bond. Such conformational preferences have been also observed for other sulfonamides and give rise to an optimal n_N_-σ*_S,C_ interaction [31]. Further notable features are the interactions between the molecules in the crystal involving the sulfonamide nitrogen. Together with the hydrogen attached to this atom (H1C) it acts as a two-fold hydrogen-bond donor towards the sulfonyl oxygen (O1) of a neighbouring molecule with an O···H distance of 2.37(2) Å (N1···O1’: 3.015(2) Å) and an N–H···O angle of 142(2)° (Figure 2B). The second contact of the NH-group involves the fluorine atom (F1) of another neighbouring molecule with an F···H distance of 2.70(2) Å (N1···F1’: 3.291(2) Å) and an N–H···F angle of 135(2)°, which can be interpreted as weak hydrogen bond. Although covalently bound fluorine is commonly considered as a poor hydrogen-bond acceptor [33–34], it tends to participate in multipolar contacts including hydrogen bonds [35], as observed herein. To our knowledge, all sulfonamides shown in Scheme 2 and Scheme 3 have not been described so far.

**Table 1:** Crystal data and structure-refinement parameters for compound **18**.

Crystal data	

Formula	C_9_H_11_FN_2_O_4_S
Formula weight	262.26 g·mol^−1^
Temperature	173(2) K
Wavelength	0.71073 Å
Crystal system	monoclinic
Space group	*P*2_1_/*n*
Unit cell dimensions	*a* = 10.7639(7) Å
	*b* = 5.2066(4) Å
	*c* = 19.532(1) Å
	β = 91.748(4)°
Volume	1094.1(1) Å^3^
*Z*	4
Density (calcd.)	1.592 g·cm^−3^
Absorption coefficient	0.316 mm^−1^
F(000)	544
Crystal size	0.26 × 0.14 × 0.05 mm^3^
Meas. Range, 2θ_max_	59.02
	
Refinement	

Refinement method	Full-matrix least- squares on *F*^2^
Data/restraints/param.	3047 / 0 / 159
Goodness-of-fit on *F*^2^	1.030
Final *R* indices	*R_1_* = 0.0402
[*I* > 2σ(*I*)]	*wR_2_* = 0.0952
*R* indices (all data)	*R_1_* = 0.0613
	*wR_2_* = 0.1031
Largest diff. peak/hole	0.386/−0.328 e·Å^−3^

### Radiochemistry

**Figure 3:**
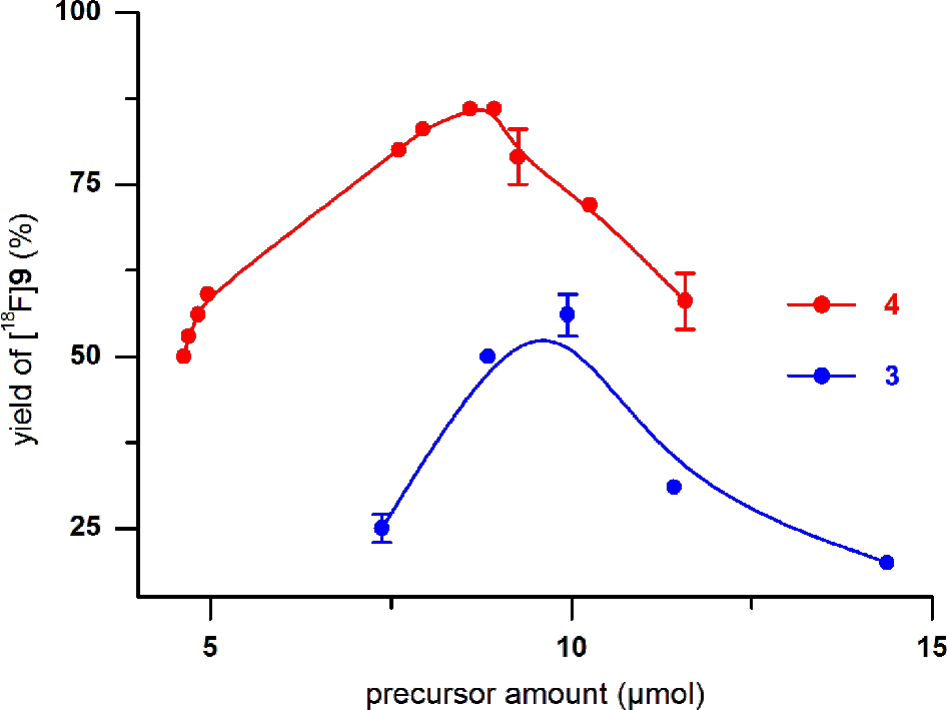
Dependence of the labelling yields of [^18^F]**9** on the precursor amount. Reactions of tosylate **3** and nosylate precursor **4** with [^18^F]F-Kryptofix_222_-carbonate complex were carried out in 500 µL of acetonitrile at 82 °C for 15 min.

The sulfonate precursor molecules **3** and **4** were subjected to nucleophilic substitution with [^18^F]fluoride. The reaction parameters (amount of precursor substance, solvent, volume, temperature, time) were carefully optimised. The following conditions were found to be optimal for the formation of radiolabelled thiocyanate [^18^F]**9**: 2.5 to 3.0 mg of **3** or **4** in 0.3–0.5 mL of acetonitrile, 82 °C, 15 min. Thermal heating was advantageous compared to microwave irradiation (up to 50 W for 7 min, 75 °C (CH_3_CN), 100 °C (DMF)). Higher labelling yields were achieved when the nosyl precursor **4** was used instead of tosylate **3** (75–85% (*n* = 12) versus 45–55% (*n* = 9), respectively; Figure 3). Notably, employing DMF as solvent resulted in significantly lower yields.

**Figure 4:**
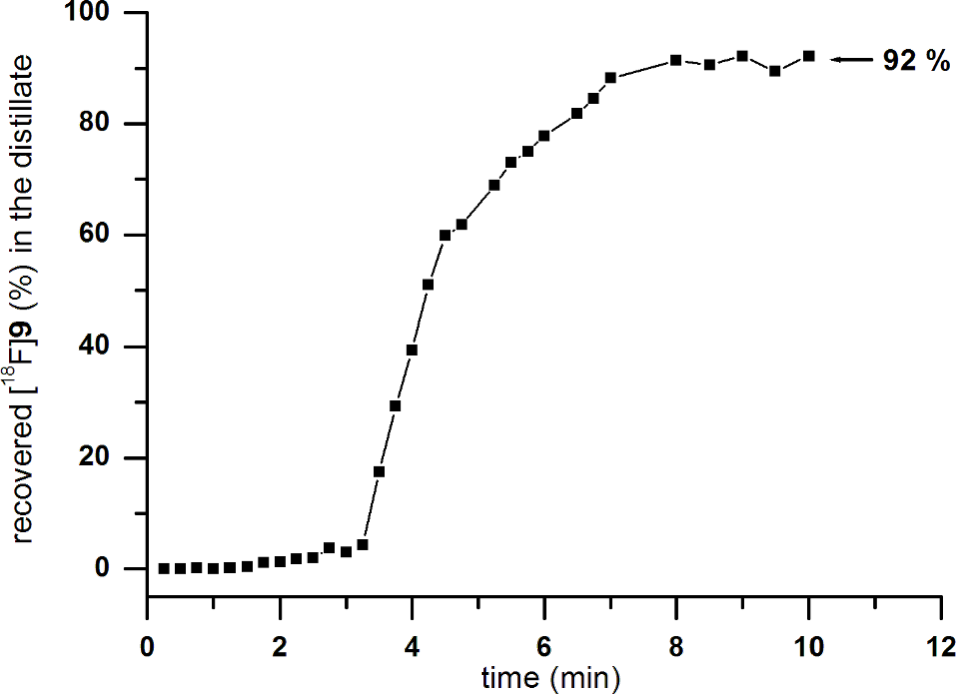
Time course of the distillation of 3-[^18^F]fluoropropyl thiocyanate ([^18^F]**9**) in the argon stream. For calculation of the distillation yield only the fraction of [^18^F]**9** in the crude mixture is considered.

The crude reaction mixture containing [^18^F]**9** was of sufficient radiochemical purity to be subjected to the next step. Alternatively, it can be isolated by distillation at atmospheric pressure in the argon stream at 80 °C and frozen out in a cooling trap at −60 °C within 10 min and a recovery of 92% (Figure 4). To transform the ^18^F-labelled thiocyanate [^18^F]**9** into the corresponding sulfonyl chloride [^18^F]**10**, [^18^F]**9** was adsorbed by a C_18_-SPE-cartridge and repetitively treated with a saturated solution of chlorine in water (prepared immediately before use). In this way 3-[^18^F]fluoropropanesulfonyl chloride ([^18^F]**10**) was obtained in radiochemical purities of 90–95% and overall decay-corrected radiochemical yields of 40–45%, within 70 min of synthesis time. Attempts to obtain [^18^F]**10** by using chlorine generated in situ from calcium hypochlorite (in 2 M HCl) were less efficient. After careful removal of excessive chlorine from the cartridge in the argon stream, we could isolate [^18^F]**10** by elution with dichloromethane, and the remaining water was removed by passing the resulting solution through a Na_2_SO_4_-filled cartridge.

**Scheme 4:**
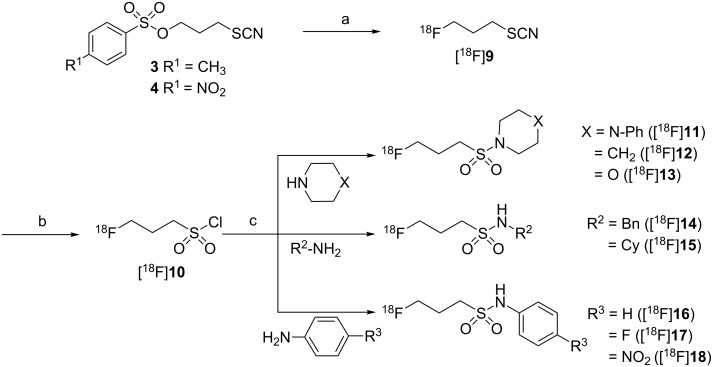
Radiosynthesis of 3-[^18^F]fluoropropanesulfonamides [^18^F]**11**–[^18^F]**18**. Reagents and conditions: (a) [^18^F]F^−^, Kryptofix_222_, K_2_CO_3_, CH_3_CN, 82 °C; (b) Cl_2_/H_2_O, C_18_-modified silica gel; (c) auxiliary base as specified in Table 2, CH_2_Cl_2_, rt.

**Figure 5:**
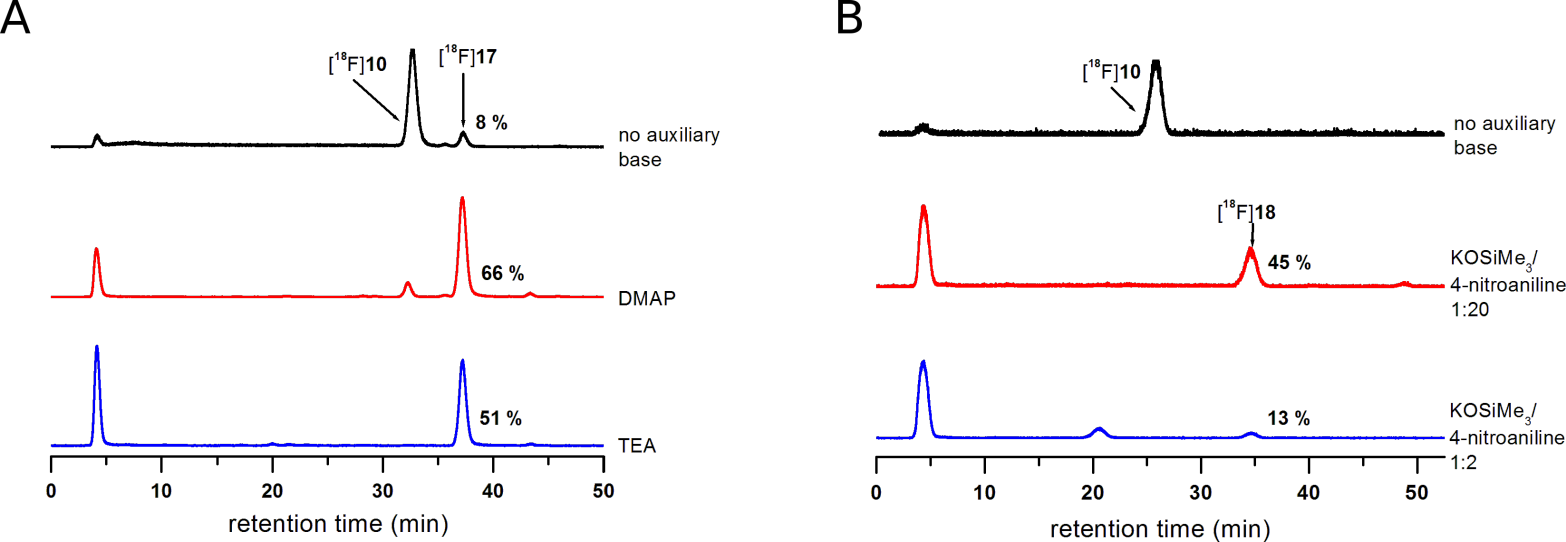
Radio-HPLC chromatograms for the reaction of [^18^F]**10** with (A) 4-fluoroaniline in the absence and presence of 4-*N*,*N*-dimethylaminopyridine (DMAP) and triethylamine (TEA) and (B) 4-nitroaniline in the absence and presence of potassium trimethylsilanolate in varying amounts relative to the nucleophile. The ordinates (counts of ^18^F-activity in arbitrary units) are omitted for clarity.

3-[^18^F]Fluoropropanesulfonyl chloride ([^18^F]**10**) was reacted with different primary and secondary aliphatic as well as primary aromatic amines (Scheme 4) at room temperature. The use of dichloromethane as solvent was superior compared to mixtures of acetonitrile and water. The reactions were carried out in the absence of any additive or with stoichiometric amounts of triethylamine (TEA) or 4-dimethylaminopyridine (DMAP) as auxiliary bases (Table 2). The radiochemical yields of the ^18^F-labelled sulfonamides [^18^F]**11**–[^18^F]**15** derived from aliphatic amines did not improve or even become attenuated upon addition of TEA or DMAP. Obviously, the aliphatic amines are sufficiently nucleophilic to undergo sulfonylation readily within short reaction times of 2–3 min. In contrast, the presence of these agents proved to be beneficial for the reaction of [^18^F]**10** with aniline and 4-fluoroaniline (Table 2 and Figure 5A) and the well known acylation catalyst DMAP was somewhat advantageous over TEA. Nevertheless, the radiochemical yields for the formation of the 4-nitroaniline-derived sulfonamide [^18^F]**18** were below 10%, even in the presence of TEA and DMAP. This can be attributed to the poor nucleophilicity of the amino group of 4-nitroaniline which is by far lower than that of aniline and 4-fluoroaniline. The difference in the nucleophilicity of these three aromatic amines is reflected by the p*K*_a_ values of their corresponding ammonium ions decreasing from 4.65 over 4.58 to 1.02 for 4-fluoroaniline, aniline, and 4-nitroaniline, respectively [36]. To achieve satisfactory conversion of the ^18^F-labelled sulfonyl chloride [^18^F]**10** with 4-nitroaniline, potassium trimethylsilanolate was tested as auxiliary base. This reagent has good solubility in organic solvents and was suggested by Laganis and Chenard as equivalent for the O^2−^ ion and is typically used to convert carboxylic esters to the corresponding potassium carboxylates under mild anhydrous conditions [37]. Its successful use for the acceleration of *O*-sulfonylations was recently described by Musachio et al. [38]. Conversion of [^18^F]**10** with 4-nitroaniline in the presence of potassium trimethylsilanolate led to the formation of the desired ^18^F-labelled sulfonamide [^18^F]**18** in radiochemical yields as high as 45%. Interestingly, a ratio of potassium trimethylsilanolate to 4-nitroaniline of 1:20 was considerably more efficient than ratios of 1:4 and 1:2 (Table 2 and Figure 5B). In this way, we could even accomplish the labelling of weakly nucleophilic amines such as 4-nitroaniline. The reason for the beneficial effect of potassium trimethylsilanolate on the sulfonylation reaction could be a partial deprotonation of the 4-nitroaniline as the basicity of siloxides is comparable to that of alkoxides [39]. A p*K*_a_ value of 21 has been reported for the amino group in 4-nitroaniline [40].

**Table 2:** Reaction of [^18^F]**10** with various aliphatic and aromatic amines (*n* ≥ 2). RCYs were determined by radio-TLC and refer to the fraction of the product related to the total ^18^F-activity.

^18^F-labelled sulfonamide	amine	RCY (%)					

		no auxiliary base	TEA	DMAP	KOSiMe_3_		
					1:2^a^	1:4^a^	1:20^a^

[^18^F]**11**	phenylpiperazine	88–89	87	72–81	—	—	—
[^18^F]**12**	piperidine	82–84	82	70–83	—	—	—
[^18^F]**13**	morpholine	77–84	76–82	77–82	—	—	—
[^18^F]**14**	benzylamine	86	74	71	—	—	—
[^18^F]**15**	cyclohexylamine	85	7	71	—	—	—
[^18^F]**16**	aniline	7	50	58	—	12–20	—
[^18^F]**17**	4-fluoroaniline	4–8	56	65	25	30–35	—
[^18^F]**18**	4-nitroaniline	<1	3	4–6	10–16	25–30	44–45

^a^Ratio of potassium trimethylsilanolate to amine.

### Stability against enzymatic degradation

Many compounds that are able to address biomolecular targets of interest for molecular imaging contain amino-substituted aromatic and heteroaromatic moieties. For the convenient radiolabelling of these molecules with fluorine-18 an ^18^F-fluoroacetylation seems to be convenient from a chemical point of view. However, ^18^F-labelled aromatic fluoroacetamides turned out to be unstable in vivo undergoing *N*-defluoroacetylation [41]. Nothing has been stated regarding the enzymes catalysing this metabolic transformation but the involvement of carboxylesterase (EC 3.1.1.1) is probable even though the participation of other hydrolases cannot be excluded [42–43]. Carboxylesterase belongs to the large class of α/β serine hydrolases, is located in the lumen of the endoplasmic reticulum of cells in many tissues, and is highly expressed in liver cells [44]. Beside its esterase activity the enzyme shows also amidase activity towards amides with various acyl chains and plays a prominent role in the hydrolytic metabolism of many drug molecules including radiopharmaceuticals [45–46]. Notably, the amidase activity of carboxylesterase is restricted to amides derived from aromatic amines [47]. This catalytic activity is crucial for the bioactivation of the acetanilide class of analgesic agents represented by paracetamol as the most important member [48]. Metabolic instability was also observed for aliphatic ^18^F-labelled fluoroacetamides [13,49]. Their fate seems to be different from their aromatic counterparts in the way that they undergo defluorination at the α-methylene group rather than hydrolytic cleavage of the amide bond [13].

The metabolic hydrolysis of sulfonamide bonds has not been reported so far. Thus, this type of chemical function can be considered as metabolically inert [42]. To support this and to assess the metabolic stability of 3-fluoropropanesulfonamides in comparison to their fluoroacetamide analogues, *N*-(4-fluorophenyl)-3-fluoropropane-1-sulfonamide (**17**) and *N*-(4-fluorophenyl)-fluoroacetamide (**19**, see Supporting Information File 1) were exposed to pig-liver esterase (PLE), the porcine homologue of carboxylesterase, in buffered aqueous solution.

Fluoroacetamide **19** was prepared by reacting 4-fluoroaniline with fluoroacetyl chloride. The activity of the enzyme preparation was verified using the chromogenic standard substrate 4-nitrophenyl butyrate in a spectrophotometric assay. The concentrations of **17** and **19** were monitored by RP-HPLC.

**Figure 6:**
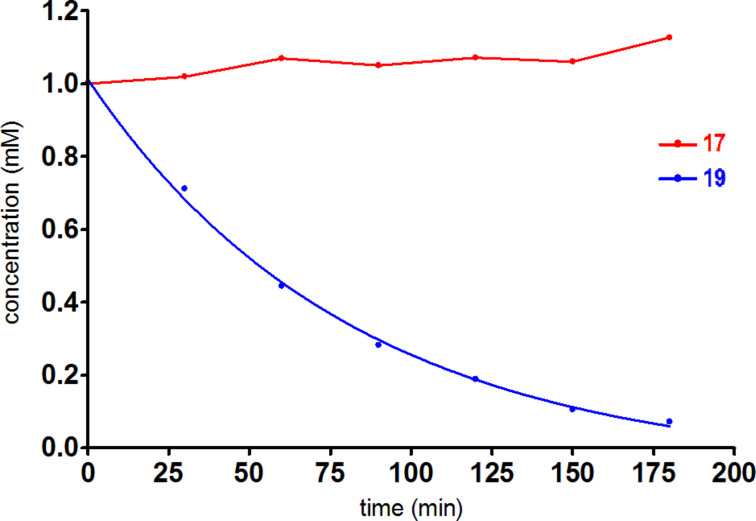
Time course of the carboxylesterase-catalysed degradation of 3-fluoropropansulfonamide **17** (red) and fluoroacetamide **19** (blue). The pseudo first-order rate constant for the decay of **19** was (0.012 ± 0.001) min^−1^ corresponding to a half-life of 57.8 min. Data points represent average values from two measurements originating from two independent experiments with SEM values less than 10% of the mean values.

As expected, sulfonamide **17** proved to be stable against degradation by carboxylesterase (Figure 6). Under the same conditions, fluoroacetamide **19** underwent degradation with a pseudo-first-order rate constant of 0.012 min^−1^ corresponding to a half-life of 58 min at an enzyme concentration of 1.4 mg/mL. This result demonstrates that the degradation of aromatic fluoroacetamides in vivo can be mediated by carboxylesterase. However, other hydrolases such as arylacetamide deacetylase might be involved in this process [43] and the nonhydrolytic disintegration of the fluoroacetyl moiety catalysed by other enzymes should be considered as well.

## Conclusion

The radiosynthesis of 3-[^18^F]fluoropropanesulfonyl chloride ([^18^F]**10**) has been optimised with regard to the preparation of the labelling precursor as well as the conditions for its efficient radiofluorination and subsequent transformation to the radiolabelled sulfonyl chloride.

A variety of primary and secondary aliphatic as well as aromatic amines were studied with respect to their reaction with [^18^F]**10** and the identity of the resulting sulfonamides was confirmed with the aid of the corresponding nonradioactive reference compounds. For one of these, *N*-(4-nitrophenyl)-3-fluoropropane-1-sulfonamide (**18**), the single-crystal X-ray structure was determined. The formation of ^18^F-labelled sulfonamides derived from aliphatic amines did not require the addition of auxiliary bases, whereas the radiochemical yields for aromatic sulfonamides were generally low without their addition. By trying different auxiliary bases it was possible to convert even electron-deficient aromatic amines, such as 4-nitroaniline, to the corresponding ^18^F-labelled sulfonamides in satisfactory yields.

The carboxylesterase-catalysed hydrolysis of an aromatic fluoroacetamide was demonstrated for the first time, implicating a radiopharmacological advantage for the 3-[^18^F]fluoropropanesulfonamides over the corresponding [^18^F]fluoroacetamides for their use in PET imaging.

## Supporting Information

File 1 Experimental procedures, characterisation data of synthesised compounds and supplementary graphical material.
