# Supplementary material for: Use of 3-[18F]fluoropropanesulfonyl chloride as a prosthetic agent for the radiolabelling of amines: Investigation of precursor molecules, labelling conditions and enzymatic stability of the corresponding sulfonamides
Source: Beilstein J Org Chem. 2013 May 27;9:1002–11. doi: 10.3762/bjoc.9.115 (PMC3678395; doi:10.3762/bjoc.9.115)
Supplement: File 1 — Experimental procedures, characterisation data of synthesised compounds and supplementary graphical material. [file Beilstein_J_Org_Chem-09-1002-s001.pdf]

Supporting Information

for

Use of 3-[^{18}F]fluoropropanesulfonyl chloride as a prosthetic agent for the radiolabelling of amines: Investigation of precursor molecules, labelling conditions and enzymatic stability of the corresponding sulfonamides

Reik Löser*[1,2], Steffen Fischer[3], Achim Hiller[3], Martin Köckerling[4], Uta Funke[3], Aurélie Maisonial[3], Peter Brus[3] and Jörg Steinbach[1,2,3]

Address: [1]Institute of Radiopharmaceutical Cancer Research (formerly Institute of Radiopharmacy), Helmholtz-Zentrum Dresden-Rossendorf (HZDR), Bautzner Landstraße 400, 01328 Dresden, Germany, [2]Department of Chemistry and Food Chemistry, Technical University of Dresden, Bergstraße 66c, 01062 Dresden, Germany, [3]Institute of Radiopharmaceutical Cancer Research, HZDR Research Site Leipzig, Permoserstraße 15, 04318 Leipzig, Germany and [4]Institute of Chemistry, University of Rostock, Inorganic Solid-State Chemistry Group, Albert-Einstein-Straße 3a, 18059 Rostock, Germany

Email: Reik Löser* - r.loeser@hzdr.de

* Corresponding author

Experimental procedures, characterisation data of synthesised compounds and supplementary graphical material

Contents

1. General remarks S3
2. Experimental procedures and characterisation data for synthesised compounds S4
3. Radiochemistry S15
4. Stability studies for enzymatic degradation S19
5. X-ray structure determination S21
6. References S21

1. General remarks

All commercial reagents and solvents were used without further purification unless otherwise specified. 3-Fluoropropanesulfonyl chloride (**10**) was purchased from Hande Sciences (Suzhou, China), all other chemicals were obtained from common suppliers. Melting points were determined on a Galen III Boetius apparatus from Cambridge Instruments. Nuclear magnetic resonance spectra were recorded on a Varian Unity 400 MHz, or Varian Gemini 300 MHz or a Mercury-VX 400 MHz spectrometer. NMR chemical shifts were referenced with the residual solvent resonances relative to tetramethylsilane (TMS). High-resolution mass spectra were obtained on a Bruker BIOAPEX 2 FTICR ESI device (7.4 Tesla; Software XMASS 7.0). Elemental analysis was performed on a LECO CHNS-932 apparatus.

Preparative column chromatography was carried out using Merck silica gel (mesh size 230–400 ASTM) with solvent mixtures as specified for the particular compounds. Preparative HPLC was performed on a Varian Prepstar system equipped with UV detector (Prostar, Varian) and automatic fraction collector Foxy 200 at a flow rate of 10 mL/min. A Microsorb C18 60-8 column (Varian Dynamax 250 × 21.4 mm) was used as the stationary phase and a binary gradient system of 0.1% CF_3COOH/water (solvent A) and 0.1% CF_3COOH/CH_3CN (solvent B) served as the eluent. The conditions for the gradient elution are specified below. Thin-layer chromatography (TLC) was performed on Merck silica gel F-254 aluminium plates with visualisation under UV (254 nm). Radio-TLC was conducted the same way with petrolether/ethyl acetate 1:2 as eluent. Radioactive spots were visualised by radio-luminescence recording using a BAS-1800 II system (Bioimaging Analyzer, Fuji Film, Japan) and images were evaluated with AIDA 2.31 software (Raytest, Germany). The identity of the radioactive spots was proven by comparison with the corresponding nonradioactive reference compounds that were spotted with radioactivity after visualisation under UV.

Analytical HPLC methods for both analysis of radiochemical reactions as well as stability assays outlined below were run by the same technical equipment consisting of a Merck Hitachi L7100 gradient pump combined with a Jasco DG2080 4-line degasser with UV detection by a Merck Hitachi L7450 diode array detector and detection of γ-radiation by a GABI detector (Raytest, Germany). The system was operated by the D-700 HSM software using a Merck Hitachi D7000 interface.

Analytical radio-HPLC was performed in gradient mode using a Multospher 120 RP18-AQ column (250 × 4.6 mm, particle size 7 µm; CS Chromatographie Service, Germany) (i) with or (ii) without pre-column (Multospher 20 × 4.6 mm, 5 µm); eluent: 5% CH_3CN/20 mM ammonium acetate (solvent A), 80% CH_3CN/20 mM NH_4OAc (solvent B); elution: 100%

solvent A (0–5 min), gradient to 100% B within 40 min, flow rate 1.0 mL/min. Separation via semi-preparative HPLC was conducted with isocratic mode using a Multispher 120 RP18-AQ column (150 × 10 mm, 5 µm; eluent: varying between 40% and 52% CH_3CN + 20 mM NH_4OAc depending on the respective sulfonamide; flow rate 1.0 mL/min).

Analytical HPLC to investigate the stability of compounds **17** and **19** towards carboxylesterase-catalyzed hydrolysis was done on a Luna C18 5 µm column (Phenomenex, 250 × 4.6 mm) using 50% CH_3CN/water containing 0.1% CF_3COOH as isocratic eluent at a flow rate of 1 mL/min.

2. Experimental procedures and characterisation data for synthesised compounds

3-Hydroxypropyl thiocyanate (2)

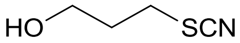

To a solution of 3-bromopropanol (**1**; 2.00 g, 14.4 mmol) in methanol (25 mL) was added potassium thiocyanate (1.54 g, 15.8 mmol) as a solid and the resulting solution was heated under reflux for 3 h. The solvent was removed from the suspension in vacuo and the residue partitioned between dichloromethane (20 mL) and water (10 mL). The aqueous phase was extracted with dichloromethane (2 × 20 mL) and the combined organic layers were washed with saturated NaCl (10 mL), dried over Na_2SO_4 and evaporated to yield **2** (1.58 g, 94%) as a colourless oil that according to ^{1}H NMR contained ca. 25% of unconverted **1**, which was judged as sufficiently pure for further steps. ^{1}H NMR (300 MHz, $CDCl_3$): δ (ppm) = 1.96–2.11 (m, 2H, $CH_2CH_2CH_2$), 3.09 (t, ^3J = 7.0 Hz, 2H, CH_2SCN), 3.78 (t, ^3J = 5.8 Hz, 2H, CH_2OH); ^{13}C NMR (75 MHz, $CDCl_3$): δ (ppm) = 30.82, 32.37 (CH_2SCN, $CH_2CH_2CH_2$), 59.71 (CH_2OH), 112.62 (SCN), in agreement with published data [1].

3-Thiocyanatopropyl tosylate (3)

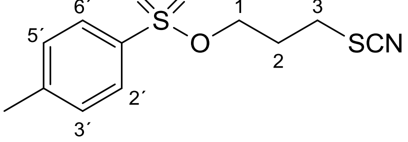

To a solution of 3-hydroxypropyl thiocyanate (**2**; 3.32 g, 28.3 mmol) in methanol (20 mL) was added tosyl chloride (5.40 g, 28.3 mmol) as solid followed by diisopropylethylamine (4.95 mL, 28.3 mmol) and 4-*N*,*N*-dimethylaminopyridine (0.17 g, 1.42 mmol). The solution was stirred for 24 h at room temperature. As the conversion of **2** was not complete, a further

portion of tosyl chloride (2.70 g, 14.1 mmol) was added. After stirring for an additional 5 h, the solvent was removed in vacuo and the residue dissolved in ethyl acetate. The solution was washed with 10% $KHSO_4$ (2 × 10 mL), water (10 mL), and saturated $NaHCO_3$, dried over Na_2SO_4, and evaporated to yield 1.23 g of a brown oil. The crude product was subjected twice (for complete removal of tosyl chloride) to column chromatography (silica gel, eluent petrol ether/ethyl acetate 3:1) to yield **3** (0.46 g, 6%) as slightly brown oil. ^{1}H NMR (300 MHz, $CDCl_3$): δ (ppm) = 2.14–2.22 (m, 2H, $CH_2CH_2CH_2$), 2.46 (s, 3H, CH_3), 3.01 (t, ^3J = 6.9 Hz, 2H, CH_2SCN), 4.18 (t, ^3J = 5.6 Hz, 2H, CH_2OTs), 7.37 (d, ^3J = 8.1 Hz, 2H, 3'-H, 5'-H), 7.79 (d, ^3J = 8.3 Hz, 2H, 2'-H, 6'-H); ^{13}C NMR (75 MHz, $CDCl_3$): δ (ppm) = 21.83 (CH_3), 29.25, 30.03 ($\mathit{C}H_2SCN$, $CH_2\mathit{C}H_2CH_2$), 66.98 (CH_2OTs), 111.50 (SCN), 128.07 (C-2', C-6'), 130.20 (C-3', C-5'), 132.65 (C-4'), 145.44 (C-1').

*Preparation of **3** from **7** and silver tosylate.* To a solution of **7** (940 mg, 4.14 mmol) in CH_3CN (25 mL) was added silver tosylate (1.62 mg, 5.8 mmol) as a solid. The resulting solution was stirred for 27 h at room temperature under the exclusion of light. The precipitate (AgI) was filtered off and washed with CH_2Cl_2 (50 mL). The combined filtrates were washed with H_2O (20 mL) and saturated NaCl (20 mL), dried over Na_2SO_4 and evaporated in vacuo to obtain an oily residue, which was purified by column chromatography (eluent petrol ether/ethyl acetate 3:1) to yield **3** (320 mg, 28% over two steps) as a nearly colourless oil that solidified after about 1 month upon treatment with a spatula. Mp 39–40 °C, ^{1}H NMR (400 MHz, $CDCl_3$): δ (ppm) = 2.13–2.23 (m, $CH_2CH_2CH_2$), 3.01 (t, ^3J = 6.9 Hz, 2H), CH_2SCN); 2.47 (s, 3H, CH_3), 4.18 (t, ^3J = 5.6 Hz, 2H, CH_2OTs), 7.37 (d, ^3J = 8.0 Hz, 2H, 3'-H, 5'-H), 7.79 (d, ^3J = 8.3 Hz, 2H, 2'-H, 6'-H), in agreement with published data [2], elemental analysis $C_{11}H_{13}NO_3S_2$ calcd. C 48.69, H 4.83, N 5.16, S 23.63, found C 48.72, H 4.79, N 5.08, S 24.22.

3-Thiocyanatopropyl nosylate (4)

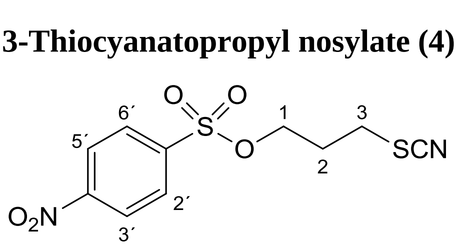

To a solution of nosyl chloride (1.55 g, 6.99 mmol) in CH_3CN (20 mL) was added Ag_2O (3.00 g, 12.93 mmol) as solid. The resulting suspension was stirred for 3 d at room temperature under the exclusion of light. The solid material was filtered off and washed with CH_3CN (15 mL). The combined filtrates were evaporated in vacuo to obtain an off-white solid. After drying under an oil pump vacuum for several hours 2.15 g (99%) of silver nosylate were yielded. *m/z* (ESI+) 107.1 ($^{107}Ag^+$), 109.1 ($^{109}Ag^+$), 148.1 ($[^{107}Ag+CH_3CN]^+$),

150.1 ([^{109}Ag+CH_3CN]$^+$), 189.1 ([^{107}Ag+2CH_3CN]$^+$), 191.1 ([^{109}Ag+2CH_3CN]$^+$); *m*/*z* (ESI-) 202.1 (*p*-$NO_2PhSO_3^-$).

To a solution of **7** (0.47 g, 2.07 mmol) in CH_3CN (10 mL) was added silver nosylate (899 mg, 2.9 mmol) obtained by the procedure above as a solid. All other steps were carried out in analogy to the preparation of **3** from **7**. Compound **4** (0.10 g, 22%) was yielded as an off-white crystalline solid after purification by column chromatography (eluent petrol ether/ethyl acetate 3:1). Mp 86 °C; ^{1}H NMR (400 MHz, $CDCl_3$): δ (ppm) 2.22–2.30 (m, 2H, $CH_2C\mathit{H}_2CH_2$), 3.05 (t, ^3J = 6.9 Hz, 2H, CH_2SCN), 4.32 (t, ^3J = 5.7 Hz, 2H, CH_2ONs), 8.13 (d, ^3J = 8.6 Hz, 2H, 3'-H, 5'-H), 8.43 (d, ^3J = 8.6 Hz, 2H, 2'-H, 6'-H); ^{13}C NMR (100 MHz, $CDCl_3$): δ (ppm) = 29.34, 29.84 (*C*H_2SCN, CH_2*C*H_2CH_2), 68.13 (CH_2ONs), 111.23 (SCN), 124.82 (C-2', C-6'), 129.44 (C-3', C-5'), 141.44 (C-1'), 151.14 (C-4'). elemental analysis $C_{10}H_{10}N_2O_5S_2$, calcd. C 39.73, H 3.33, N 9.27 S 21.21, found C 40.27, H 3.49, N 9.02, S 20.77.

*Preparation of **4** by nosylation of **2***. To a solution of **2** (1.51 g, 12.9 mmol) and nosyl chloride (3.58 g, 16.13 mmol) in THF (15 mL) was added potassium trimethylsilanolate (4.14 g, 32.25 mmol) as solid under ice cooling. After stirring for 2 h at 4 °C, H_2O (100 mL) and CH_2Cl_2 (50 mL) were added. The aqueous layer was separated and extracted with CH_2Cl_2 (2 × 50 mL). The combined organic phases were washed with H_2O (30 mL) and saturated NaCl (30 mL), dried over Na_2SO_4 and evaporated in vacuo. The obtained oily residue was purified by column chromatography (eluent petrol ether/ethyl acetate 3:1) to obtain **4** (480 mg, 13%) as an off-white crystalline solid. The analytical data are identical to those from above.

3-Chloropropyl thiocyanate (6)

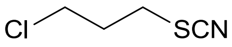

To a solution of 1-bromo-3-chloropropane (**5**; 2.00 g, 12.8 mmol) in methanol (20 mL) was added potassium thiocyanate (1.24 g, 12.8 mmol) as a solid and the resulting solution was heated under reflux for 6 h. The solvent was removed from the suspension (white precipitate of potassium bromide) in vacuo. The obtained residue was suspended in dichloromethane (30 mL) and washed with water (20 mL). The aqueous layer was extracted with dichloromethane (2 × 20 mL). The combined organic phases were washed with saturated NaCl (10 mL), dried over Na_2SO_4 and evaporated in vacuo to yield 1.43 g of a nearly colourless oil. The crude product was purified by vacuum distillation (95 °C, 3 mbar; Lit. 109 °C, 10 Torr, [3]; 115 °C 12 Torr, [4]) to yield **6** (0.63 g, 37%) as a colourless oil. ^{1}H NMR (400 MHz, $CDCl_3$): δ (ppm) = 2.25–2.32 (m, 2H, $CH_2C\mathit{H}_2CH_2$), 3.13 (t, ^3J = 6.8 Hz,

2H, CH_2SCN), 3.71 (t, ^3J = 6.0 Hz, CH_2Cl); ^{13}C NMR (100 MHz, $CDCl_3$): δ (ppm) = 30.97, 32.10 (CH_2SCN, $CH_2CH_2CH_2$), 41.96 (CH_2Cl), 111.66 (SCN).

3-Iodopropyl thiocyanate (7)

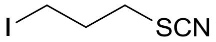

To a solution of **6** (2.0 g, 14.8 mmol) in acetone (30 mL) was added sodium iodide (11.09 g, 74.0 mmol) as solid. The resulting suspension was heated under reflux for 24 h and the solvent partially removed in vacuo. Water (40 mL) was added to the residue and the mixture was extracted with dichloromethane (3 × 50 mL). The combined organic layers were washed with brine (30 mL), dried over sodium sulfate and evaporated in vacuo to obtain 3.24 g of a yellow oil that was fractionated by vacuum distillation (Kugelrohr apparatus; 95 °C, 0.20–0.30 mbar) to yield **7** (0.82 g, 24%) as a slightly yellow oil. ^1H NMR (400 MHz, $CDCl_3$): δ (ppm) = 2.28–2.35 (m, 2H, $CH_2CH_2CH_2$), 3.09 (t, ^3J = 6.8 Hz, 2H, CH_2SCN), 3.32 (t, ^3J = 6.4 Hz, CH_2I); ^{13}C NMR (100 MHz, $CDCl_3$): δ (ppm) = 2.49 (CH_2I), 32.58, 34.55 (CH_2SCN, $CH_2CH_2CH_2$), 111.56 (SCN); elemental analysis C_4H_6INS calcd. C 21.16, H 2.66 N 6.17, S 14.12, found C 20.16, H 2.53, N 5.76, S 13.14.

*Preparation of **7** from **2** by Appel-type reaction.* To a solution of triphenylphosphine (1.23 g, 4.70 mmol) in THF (10 mL) was added iodine (1.08 g, 4.27 mmol) as a solid in portions. A brown precipitate was formed and imidazole (0.58 g, 8.54 mmol) was added as a solid upon which the precipitate dissolved. Subsequently, a solution of 3-hydroxypropyl thiocyanate (**2**; 0.50 g, 4.27 mmol) in THF (5 mL) was added under ice cooling whereupon the brown colour of the solution vanished. After stirring for 1 h at room temperature the reaction mixture was diluted with water (20 mL) and extracted with dichloromethane (3 × 20 mL). The combined organic layers were washed with 10% $Na_2S_2O_3$ (20 mL) and saturated NaCl (20 mL) and dried over Na_2SO_4. After removing the solvent in vacuo a partly solid residue (containing triphenylphosphine oxide as the solid component) was obtained that was suspended in petrol ether/ethyl acetate 4:1. The resulting mixture was filtered, the solid residue washed with petrol ether/ethyl acetate 4:1 and the combined filtrates were concentrated in vacuo to yield 0.87 g (90%) of a yellow oil from which triphenylphosphine oxide crystallised. The crude product was used without characterisation for further conversions.

3-Fluoropropyl thiocyanate (9)

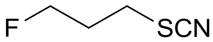

To a solution of 1-fluoro-3-iodopropane (**8**; 4.00 g, 21.3 mmol) in methanol (30 mL) was added potassium thiocyanate (2.27 g, 23.4 mmol) as a solid, and the resulting solution was heated under reflux for 3 h. The solution was concentrated in vacuo and the obtained residue suspended in dichloromethane (20 mL) and washed with water (10 mL). The aqueous layer was extracted with dichloromethane (2 × 20 mL) and the combined organic phases were dried over Na_2SO_4 and evaporated in vacuo to yield **9** (2.29 g, 90%) as a slightly brown oil, which was sufficiently pure for further conversion. ^{1}H NMR (300 MHz, $CDCl_3$): δ (ppm) = 2.14–2.32 (m, 2H, $CH_2C\mathit{H}_2CH_2$), 3.10 (t, ^3J = 7.0 Hz, 2H, CH_2SCN), 4.61 (dt, $^2J_{H,F}$ = 47.0 Hz, $^3J_{H,H}$ = 5.4 Hz, 2H, CH_2F); ^{13}C NMR (75 MHz, $CDCl_3$): δ (ppm) = 30.02 (d, $^3J_{C,F}$ = 4.3 Hz, $\mathit{C}H_2SCN$), 30.77 (d, $^2J_{C,F}$ = 20.4 Hz, $CH_2\mathit{C}H_2CH_2$), 80.87 (d, $^1J_{C,F}$ = 167.3 Hz, CH_2F), 111.79 (SCN); ^{19}F NMR (75 MHz, $CDCl_3$): δ (ppm) = −223.02 (m, $F(CH_2)_3$); compound described in [5], no spectroscopic data available.

3-Fluoropropanesulfonyl chloride (10)

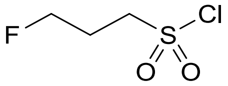

Deionised water (20 mL) was saturated with chlorine (generated from solid calcium hypochlorite and concentrated hydrochloric acid) under ice cooling. A solution of **9** (0.50 g, 4.2 mmol) in acetic acid (10 mL) was added and cooling was stopped. After stirring for 5 min (green colour of the elemental chlorine disappeared) dichloromethane (20 mL) was added. The aqueous layer was extracted with dichloromethane (2 × 20 mL) and the combined organic phases dried over Na_2SO_4. After removal of the solvent in vacuo 0.62 g of a nearly colourless oil were obtained. The crude product was purified by vacuum distillation (Kugelrohr apparatus; 95 °C, 0.82 mbar, Lit. 95.5–96 °C, 12 Torr, [6]) to yield **10** (0.21 g, 31%) as a slightly yellow oil. ^{1}H NMR (400 MHz, $CDCl_3$): δ (ppm) = 2.36–2.51 (m, 2H, $CH_2C\mathit{H}_2CH_2$), 3.83 (t, ^3J = 7.6 Hz, 2H, CH_2SO_2Cl), 4.62 (dt, $^2J_{H,F}$ = 46.8 Hz, $^3J_{H,H}$ = 5.6 Hz, 2H, CH_2F); ^{13}C NMR (100 MHz, $CDCl_3$): δ (ppm) = 25.90 (d, $^2J_{C,F}$ = 20.7 Hz, $CH_2\mathit{C}H_2CH_2$), 61.62 (d, $^3J_{C,F}$ = 4.3 Hz, CH_2SO_2Cl), 80.55 (d, $^1J_{C,F}$ = 169.0 Hz, CH_2F).

1-(3-Fluoropropanesulfonyl)-4-phenylpiperazine (11)

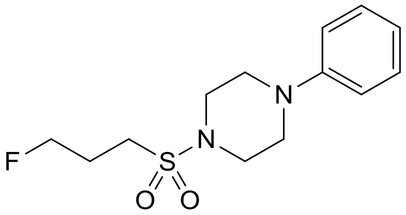

A solution of 0.3 g of crude **10** in dichloromethane (5 mL) was added to a solution of 1-phenylpiperazine (0.30 g, 1.85 mmol) in dichloromethane (5 mL) followed by triethylamine (0.19 g, 1.85 mmol). The resulting solution was heated under reflux for 2 h. After this, 1 M NaOH (10 mL) was added to the solution and the aqueous layer was extracted with dichloromethane (2 × 20 mL). The combined organic phases were dried over Na_2SO_4 and the solvent was removed in vacuo to yield 0.53 g of crude material which was subjected to column chromatograpy (silica gel, eluent dichloromethane/methanol 60:1) to obtain **11** (0.32 g, 59%) as an off-white solid. Mp 103–106 °C; ^{1}H NMR (400 MHz, $CDCl_3$): δ (ppm) = 2.18–2.33 (m, 2H, $CH_2CH_2CH_2$), 3.09 (t, ^3J = 7.6 Hz, 2H, CH_2SO_2N), 3.26 (t, ^3J = 5.0 Hz, 4H, 2 × CH_2N), 3.45 (t, ^3J = 5.0 Hz, 4H, 2 × CH_2N), 4.59 (dt, $^2J_{H,F}$ = 46.9 Hz, $^3J_{H,H}$ = 5.6 Hz, 2H, CH_2F), 6.90–6.96 (m, 3H, Ph-2'-H, Ph-6'-H, Ph-1'-H), 7.27–7.32 (m, 2H, Ph-3'-H, Ph-5'-H); ^{13}C NMR (100 MHz, $CDCl_3$): δ (ppm) = 24.71 (d, $^2J_{C,F}$ = 20.6 Hz, $CH_2CH_2CH_2$), 45.42 (d, $^3J_{C,F}$ = 4.2 Hz, CH_2SO_2N), 45.89, 49.83 (2 × CH_2N), 81.85 (d, $^1J_{C,F}$ = 167.3 Hz, CH_2F), 117.71 (C-2', C-6'), 121.09 (C-4'), 129.45 (C-3', C-5'), 150.87 (C-1'); HRMS-ESI: M + Na, calcd. 303.1173, measd. 303.1173.

4-(3-Fluoropropanesulfonyl)piperidine (12). General procedure for aliphatic sulfonamides 12–15.

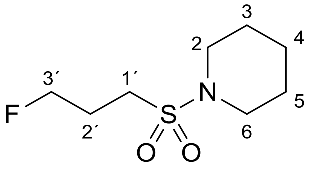

To a solution of 3-fluoropropanesulfonyl chloride (**10**; 0.05 g, 0.31 mmol) in dichloromethane (2 mL) was added piperidine (0.030 mL, 0.31 mmol) and triethylamine (0.042 mL, 0.31 mmol) and the resulting mixture was heated under reflux for 2 h. The cold solution was diluted with dichloromethane (5 mL) and washed with 1 M HCl (1 mL). The aqueous phase was extracted with dichloromethane (2 × 3 mL) and the combined organic layers washed with brine (2 mL), dried over sodium sulfate and evaporated in vacuo to obtain an oily residue that was repeatedly treated with *n*-hexane to yield **12** (42 mg, 65%) as a slightly yellow oil. ^{1}H NMR (400 MHz, $CDCl_3$): δ (ppm) = 1.53–1.69 (m, 6H, 3-H, 4-H, 5-H), 2.13–2.28 (m, 2H,

2'-H), 3.01 (t, ^3J = 7.6 Hz, 2H, CH_2SO_2N), 3.24 (t, ^3J = 5.4 Hz, 4H, CH_2N), 4.56 (dt, $^2J_{H,F}$ = 46.9 Hz, $^3J_{H,H}$ = 5.7 Hz, 2H, CH_2F); ^{13}C NMR (100 MHz, $CDCl_3$): δ (ppm) = 23.88 (C-4), 24.75 ($^2J_{C,F}$ = 20.6 Hz, C-2'), 25.76 (C-3, C-5), 45.32 (d, $^3J_{C,F}$ = 4.2 Hz, CH_2SO_2N), 46.74 (C-2, C-6), 81.98 (d, $^1J_{C,F}$ = 166.9 Hz, CH_2F); ^{19}F NMR (376 MHz, $CDCl_3$): δ (ppm) = −221.20 (m); HRMS-ESI: 2M + Na, calcd. 441.1671, measd. 441.1669.

4-(3-Fluoropropanesulfonyl)morpholine (13)

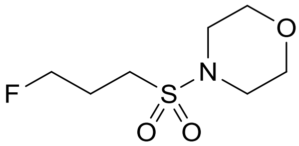

Applying the method described for **12** using morpholine (0.027 mL, 0.31 mmol) instead of piperidine led to a colourless oil that solidified in the refrigerator. Washing with *n*-hexane afforded **15** (63 mg, 97%) as a white solid. Mp 66–69 °C, ^{1}H NMR (400 MHz, $CDCl_3$): δ (ppm) = 2.16–2.31 (m, 2H, $CH_2CH_2CH_2$), 3.05 (t, ^3J = 7.6 Hz, 2H, CH_2SO_2N), 3.28 (t, ^3J = 4.8 Hz, 4H, 2 × CH_2N), 3.77 (t, ^3J = 4.8 Hz, 4H, 2 × CH_2O), 4.58 (dt, $^2J_{H,F}$ = 46.9 Hz, $^3J_{H,H}$ = 5.6 Hz, 2H, CH_2F); ^{13}C NMR (100 MHz, $CDCl_3$): δ (ppm) = 24.61 (d, $^2J_{C,F}$ = 20.6 Hz, $CH_2CH_2CH_2$), 44.97 (d, $^3J_{C,F}$ = 4.1 Hz, CH_2SO_2N), 45.94 (CH_2N), 66.69 (CH_2O), 81.82 (d, $^1J_{C,F}$ = 167.3 Hz, CH_2F); ^{19}F NMR (376 MHz, $CDCl_3$): δ (ppm) = −221.43 (m); HRMS-ESI: 2M + Na, calcd. 445.1256, measd. 445.1267.

***N*-Benzyl-3-fluoropropane-1-sulfonamide (14).**

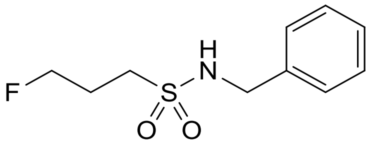

Following the procedure described for **12** using benzylamine (0.034 mL, 0.31 mmol) instead of piperidine resulted in a light brown oil that solidified upon cooling. The solid was washed with *n*-hexane and dried in the desiccator to yield 68 mg (95%) of an off-white solid. Mp 51–52 °C, ^{1}H NMR (400 MHz, $CDCl_3$): δ (ppm) = 2.06–2.25 (m, 2H, 2'-H), 3.07 (t, ^3J = 7.6 Hz, 2H, 2H, CH_2SO_2NH), 4.32 (d, ^3J = 5.9 Hz, 2H, $NHCH_2$), 4.50 (dt, $^2J_{H,F}$ = 46.9 Hz, $^3J_{H,H}$ = 5.6 Hz, 2H, CH_2F), 4.69 (br s, 1H, NH), 7.29–7.41 (m, 5H, Ph-H); ^{13}C NMR (100 MHz, $CDCl_3$): δ (ppm) = 25.27 (d, ^2J = 20.7 Hz, $CH_2CH_2CH_2$), 47.42 (s, $PhCH_2NH$), 49.72 (d, ^3J = 4.2 Hz, CH_2SONH), 81.77 (d, ^1J = 167.3 Hz, FCH2), 128.53, 128.60, 128.83, 136.81 C_{arom}; ^{19}F NMR (376 MHz, $CDCl_3$): δ (ppm) = −220.81 (m). HRMS-ESI: 2M + Na, calcd. 485.1358, measd. 485.1345.

***N*-Cyclohexyl-3-fluoropropane-1-sulfonamide (15)**

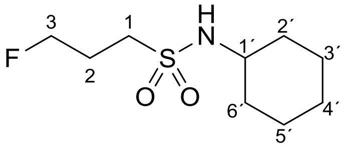

Applying the method described for **12** using cyclohexylamine (0.036 mL, 0.31 mmol) instead of piperidine afforded a light-brown oil that solidified upon cooling. The solid was washed with *n*-hexane and dried in the desiccator to yield 68 mg (98%) of an off-white solid. Mp 62–69 °C, ^{1}H NMR (400 MHz, $CDCl_3$): δ (ppm) = 1.10–1.38 (m, 5H, cyclohexyl), 1.55–1.60 (m, 1H, cyclohexyl), 1.68–1.78 (m, 2H, cyclohexyl), 1.93–2.03 (m, 2H, cyclohexyl), 2.13–2.28 (m, 2H, 2-H), 3.15 (t, ^{3}J=7.6 Hz, 2H, CH_2SO_2N), 3.23–3.35 (m, 1H, 1'-H), 4.19 (d, ^{3}J = 7.7 Hz, 1H, NH), 4.57 (dt, $^{2}J_{H,F}$ = 46.9 Hz, $^{3}J_{H,H}$ = 5.7 Hz, 2H, CH_2F); ^{13}C NMR (100 MHz, $CDCl_3$): δ (ppm) = 24.96 (C-4'), 25.27 (C-3', C-5'), 25.52 (d, $^{2}J_{C,F}$ = 20.7 Hz, C-2), 34.78 (C-2', C-6'), 45.94 (C-1'), 50.50 (d, $^{3}J_{C,F}$=4.1 Hz, CH_2SO_2N), 81.88 (d, $^{1}J_{C,F}$=167.1 Hz, CH_2F); HRMS-ESI: 2M + Na, calcd. 469.1984, measd. 469.1977.

***N*-Phenyl-3-fluoropropane-1-sulfonamide (16)**

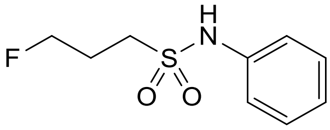

The method described for **12** was followed using aniline (0.028 mL, 0.31 mmol) instead of piperidine. The reaction mixture was heated under reflux for 5 h. The solution was diluted with dichloromethane (10 mL) and washed with 1 M HCl (3 × 2 mL), brine (1 mL), dried over sodium sulfate and evaporated in vacuo. The obtained brown residue was purified by preparative HPLC in three single runs using a gradient from 30% B to 55% B over 20 min followed by a gradient up to 95% B over 5 min. The peak with t_R = 16.4 min was collected and the combined fractions were concentrated in vacuo followed by lyophilisation of the aqueous residue to obtain **16** (23 mg, 34%) as an off-white solid. Mp 45–46 °C, ^{1}H NMR (400 MHz, $CDCl_3$): δ (ppm) = 2.15–2.30 (m, 2H, $CH_2CH_2CH_2$), 3.25 (t, ^{3}J = 7.6 Hz, 2H, CH_2SO_2NH), 4.54 (dt, $^{2}J_{H,F}$ = 46.9 Hz, $^{3}J_{H,H}$ = 5.6 Hz, 2H, CH_2F), 6.60 (s, 1H, NH), 7.17–7.25 (m, 3H, H_{arom}), 7.32–7.40 (m, 2H, H_{arom}); ^{13}C NMR (100 MHz, $CDCl_3$): δ (ppm) = 25.12 (d, $^{2}J_{C,F}$ = 20.7 Hz, $CH_2CH_2CH_2$), 48.24 (d, $^{3}J_{C,F}$=4.3 Hz, CH_2SO_2NH), 81.63 (d, $^{1}J_{C,F}$ = 167.6 Hz), 121.22 (C-2', C-6'), 125.95 (C-4'), 129.64 (C-3', C-5'), 136.51 (C-1'); ^{19}F NMR (376 MHz, $CDCl_3$): δ (ppm) = −220.79 (m); HRMS-ESI: 2M + Na, calcd. 457.1045, measd. 457.1039.

***N,N*-Bis(3-fluoropropanesulfonyl)aniline (16a)**

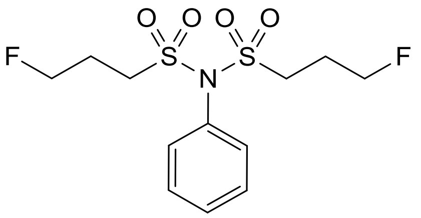

Obtained from the reaction batch for **16** by combining the fractions corresponding to the peak with t_R=24.3 min. 9 mg (9%) were yielded after evaporation in vacuo and repeated dissolving of the residue in CH_3CN (3 × 10 mL) and evaporation to remove residual H_2O. Mp 147–150 °C, ^1H NMR (400 MHz, $CDCl_3$): δ (ppm) = 2.25–2.39 (m, 4H, $CH_2CH_2CH_2$), 3.72 (t, ^3J = 7.6 Hz, 4H, CH_2SO_2N), 4.57 (dt, $^2J_{H,F}$ = 46.8 Hz, $^3J_{H,H}$ = 5.6 Hz, 4H, CH_2F), 7.36–7.42 (m, 2H, H_{arom}), 7.45–7.54 (m, 3H, H_{arom}); ^{13}C NMR (100 MHz, $CDCl_3$): δ (ppm) = 24.73 (d, $^2J_{C,F}$ = 20.9 Hz, $CH_2CH_2CH_2$), 51.92 (d, $^3J_{C,F}$ = 4.5 Hz), 81.35 ($^1J_{C,F}$ = 177.8 Hz), 129.99, 131.03, 131.26, 133.54 (C_{arom}); ^{19}F NMR (376 MHz, $CDCl_3$): δ (ppm) = −221.3 (m); HRMS-ESI: M + Na, calcd. 364.0459, measd. 364.0457.

***N*-(4-Fluorophenyl)-3-fluoropropane-1-sulfonamide (17)**

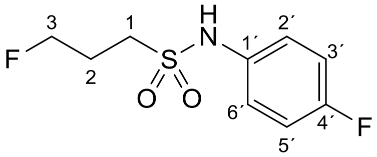

The method described for **12** was followed using 4-fluoroaniline (0.029 mL, 0.31 mmol) instead of piperidine. The reaction mixture was heated under reflux for 5 h. The solution was diluted with dichloromethane (10 mL) and washed with 1 M HCl (3 × 2 mL), brine (1 mL), dried over sodium sulfate and evaporated in vacuo. The obtained brown residue was purified by preparative HPLC in three single runs using a gradient from 40% B to 60% B over 20 min followed by a gradient up to 95% B over 5 min. The peak with t_R = 14.5 min was collected and the combined fractions were concentrated in vacuo followed by lyophilisation of the aqueous residue to obtain **17** (25 mg, 34%) as a white solid. Mp 54–56 °C, ^1H NMR (400 MHz, $CDCl_3$): δ (ppm) = 2.13–2.30 (m, 2H, $CH_2CH_2CH_2$), 3.21 (t, ^3J = 7.6 Hz, 2H, CH_2SO_2NH), 4.55 (dt, $^2J_{H,F}$=46.9 Hz, $^3J_{H,H}$=5.6 Hz, 2H, CH_2F), 6.46 (s, 1H, NH), 7.02–7.09 (m, 2H, 3'-H, 5'-H), 7.20–7.25 (m, 2H, 2'-H, 6'-H); ^{13}C NMR (100 MHz, $CDCl_3$): δ (ppm) = 25.15 (d, $^2J_{C,F}$ = 20.7 Hz, $CH_2CH_2CH_2$), 48.21 (d, $^3J_{C,F}$ = 4.1 Hz, CH_2SO_2NH), 81.62 (d, $^1J_{C,F}$ = 167.7 Hz, CH_2F), 116.73 (d, $^2J_{C,F}$ = 22.9 Hz, C-3', C-5'), 124.26 (d, $^3J_{C,F}$ = 8.3 Hz, C-2', C-6'), 132.20 (d, $^4J_{C,F}$ = 3.0 Hz, C-1'), 160.93 (d, $^1J_{C,F}$ = 246.1 Hz, C-4'); ^{19}F NMR (376 MHz, $CDCl_3$): δ (ppm) = −220.81 (m, 3-F), −116.29 (m, 4'-F); HRMS-ESI: M + Na, calcd. 258.0377; measd. 258.0370.

***N,N*-Bis(3-fluoropropanesulfonyl)-4-fluoroaniline (17a)**

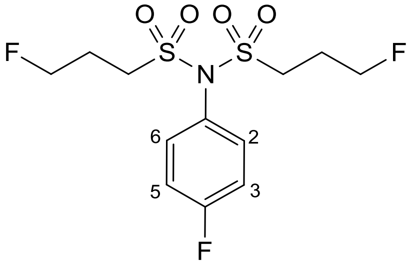

Obtained from the reaction batch for **17** by combining the fractions corresponding to the peak with t_R = 22.1 min. An amount of 9 mg (8%) was yielded after evaporation in vacuo and lyophilisation of the aqueous residue. Mp 92–96 °C, ^{1}H NMR (400 MHz, $CDCl_3$): δ (ppm) = 2.24–2.39 (m, 4H, $CH_2CH_2CH_2$), 3.71 (t, ^{3}J = 7.6 Hz, 4H, CH_2SO_2N), 4.57 (d, $^{2}J_{H,F}$ = 46.8 Hz, $^{3}J_{H,H}$ = 5.6 Hz, 4H, CH_2F), 7.16 (dd, $^{3}J_{H,H}$ = 9.0 Hz, $^{3}J_{H,F}$ = 8.0 Hz, 2H, 3-H, 5-H), 7.37 (dd, $^{3}J_{H,H}$ = 9.0 Hz, $^{3}J_{H,F}$ = 4.7 Hz, 2H, 2-H, 6-H); ^{13}C NMR (100 MHz, $CDCl_3$): δ (ppm) = 24.71 (d, $^{2}J_{C,F}$ = 20.8 Hz, $CH_2CH_2CH_2$), 51.90 (d, $^{3}J_{C,F}$ = 4.6 Hz, CH_2SO_2NH), 81.24 (d, $^{1}J_{C,F}$ = 168.6 Hz, CH_2F), 116.99 (d, $^{2}J_{C,F}$ = 23.2 Hz, C-3, C-5), 129.40 (d, $^{4}J_{C,F}$=3.4 Hz, C-1'), 133.04 (d, $^{3}J_{C,F}$ = 9.2 Hz, C-2, C-6), 163.74 (d, $^{1}J_{C,F}$ = 250.9 Hz, C-4'); ^{19}F NMR (376 MHz, $CDCl_3$): δ (ppm) = –221.36 (m, 3-F), –109.30 (m, 4'-F); HRMS-ESI: M + Na, calcd. 382.0365, measd 382.0365.

***N*-(4-Nitrophenyl)-3-fluoropropane-1-sulfonamide (18)**

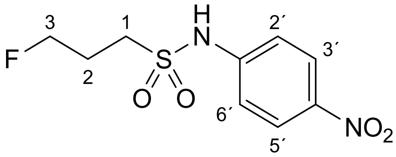

To a suspension of 4-nitroaniline (47 mg, 0.34 mmol) in dichloromethane (2 mL) was added a solution of pyridine (0.028 mL, 0.34 mmol) in dichloromethane (1 mL). The reaction mixture was stirred for 7 d at room temperature. The solvent was removed in vacuo and the obtained residue subjected to preparative HPLC using isocratic elution with 50% B as eluent. The product-containing fractions were combined and concentrated in vacuo leading to a yellow precipitate. The solid was collected by filtration and dried in a desiccator over P_4O_{10} under reduced pressure to yield **18** (18 mg, 26%) as yellow crystals. Mp 132–134 °C, ^{1}H NMR (400 MHz, DMSO-d_6): δ (ppm) = 1.98–2.13 (m, 2H, $CH_2CH_2CH_2$), 3.38 (t, ^{3}J = 7.6 Hz, 2H, CH_2SO_2NH), 4.51 (dt, $^{2}J_{H,F}$ = 47.0 Hz, $^{3}J_{H,H}$ = 6.0 Hz, 2H, CH_2F), 7.39 (d, ^{3}J = 9.6 Hz, 2H, 2'-H, 6'-H), 8.23 (d, ^{3}J = 9.2 Hz, 2H, 3'-H, 5'-H), 10.82 (s, 1H, NH); ^{13}C NMR (100 MHz, DMSO-d_6): δ (ppm) = 24.53 (d, $^{2}J_{C,F}$ = 20.6 Hz, $CH_2CH_2CH_2$), 47.74 (d, $^{3}J_{C,F}$ = 5.5 Hz, CH_2SO_2NH), 81.52 (d, $^{1}J_{C,F}$ = 163.2 Hz, CH_2F), 117.63 (C-2', C-6'), 125.51 (C-3', C-5'),

142.35 (C-4’), 144.71 (C-1’); ^{19}F NMR (376 MHz, DMSO-d_6): δ (ppm) = −218.99 (m); HRMS-ESI: M + Na, calcd. 285.0316, measd. 285.0314.

***N*,*N*-Bis(3-fluoropropanesulfonyl)-4-nitroaniline (18a)**

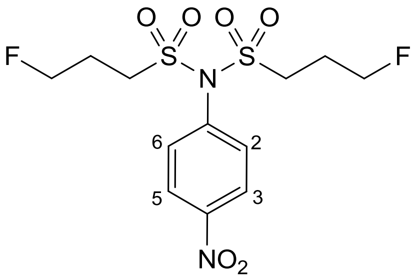

The method described for **12** was followed using 4-nitroaniline (43 mg, 0.31 mmol) instead of piperidine. TLC monitoring indicated that after heating under reflux for 5 h a considerable amount of 4-nitroaniline was still present that did not decrease upon stirring for an additional 23 h at room temperature. The reaction mixture was diluted with dichloromethane (10 mL) and washed with 1 M HCl (3 × 2 mL). The organic layer was washed with brine (1 mL), dried over sodium sulfate and evaporated in vacuo. The obtained residue (ca. 80 mg) was purified by column chromatography (silica gel, eluent petrol ether/ethyl acetate 3:1). The product-containing fractions were combined and evaporated in vacuo. The obtained residue was crystallised from dichloromethane/cyclohexane and the precipitate was collected by filtration and washed with *n*-pentane to yield **18a** (10 mg, 17%) as yellow needles. Mp 110–115 °C, ^{1}H NMR (400 MHz, $CDCl_3$): δ (ppm) = 2.23–2.39 (m, 4H, $CH_2CH_2CH_2$), 3.74 (t, ^3J = 7.6 Hz, 4H, CH_2SO_2N), 4.57 (d, $^2J_{H,F}$ = 46.7 Hz, $^3J_{H,H}$ = 5.6 Hz, 4H, CH_2F), 7.58 (d, ^3J = 9.0 Hz, 2H, 2-H, 6-H), 8.34 (^3J = 9.0 Hz, 2H, 3-H, 5-H); ^{13}C NMR (100 MHz, $CDCl_3$): δ (ppm) = 24.69 (d, $^2J_{C,F}$ = 20.9 Hz, $CH_2CH_2CH_2$), 52.28 (d, $^3J_{C,F}$ = 4.5 Hz, CH_2SO_2NH), 81.08 (d, $^1J_{C,F}$ = 169.0 Hz, CH_2F), 124.94 (C-2, C-6), 132.29 (C-3, C-5), 139.02 (C-4), 148.83 (C-1); ^{19}F NMR (376 MHz, $CDCl_3$): δ (ppm) = −221.45; HRMS-ESI: M + Na, calcd. 409.0310, measd. 409.0313.

***N*-(4-Fluorophenyl)fluoroacetamide (19)**

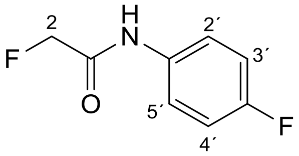

To a solution of 4-fluoroaniline (0.102 mL, 1.04 mmol) and triethylamine (0.144 mL, 1.04 mmol) in dichloromethane (5 mL) was added fluoroacetyl chloride (0.10 g, 1.04 mmol) under ice cooling followed by 4-*N*,*N*-dimethylaminopyridine (0.013 g, 0.11 mmol). After stirring for 3 h at room temperature the reaction mixture was diluted with dichloromethane (7 mL), and the solution was washed with 2 M HCl (3 × 2 mL) and brine (2 mL) and dried over sodium sulfate. After evaporation of the solvent in vacuo a solid was obtained that was recrystallised from water (5 mL) to yield **19** (83 mg, 47%) as white needles. Mp 113–117 °C (Lit. 114–115 °C [7]), ^{1}H NMR (400 MHz, DMSO-d_6): δ (ppm) = 4.98 (d, $^{2}J_{H,F}$ = 46.8 Hz, 2H, CH_2), 7.17 (ps t, $^{3}J_{H,F}$ = $^{3}J_{H,H}$ = 8.9 Hz, 3’-H, 5’-H), 7.67 (dd, $^{3}J_{H,H}$ = 9.1 Hz, $^{4}J_{H,F}$ = 5.0 Hz, 2H, 2’-H, 6’-H), 10.14 (s, 1H, NH); ^{13}C NMR (100 MHz, DMSO-d_6): δ (ppm) = 79.87 (d, $^{1}J_{C,F}$ = 179.3 Hz, CH_2), 115.29 (d, $^{2}J_{C,F}$ = 22.3 Hz, C-3’, C-5’), 121.76 (d, $^{3}J_{C,F}$ = 7.9 Hz, C-2’, C-6’), 134.37 (d, $^{4}J_{C,F}$ = 2.7 Hz, C-1’), 158.35 (d, $^{1}J_{C,F}$ = 240.4 Hz, C-4’), 165.84 (d, $^{2}J_{C,F}$ = 18.7 Hz, C=O); ^{19}F NMR (376 MHz, DMSO-d_6): δ (ppm) = −225.48 (m), −119.09 (m).

3. Radiochemistry

Processing of [^{18}F]fluoride for radiosynthesis. No-carrier-added aqueous [^{18}F]fluoride was produced in a PETtrace® 16.5 MeV cyclotron (GE Healthcare-Siemens, Germany) or a IBA CYCLONE 18/9 cyclotron by irradiation of [^{18}O]H_2O via the ^{18}O(p,n)^{18}F nuclear reaction. To prepare a reactive anhydrous [^{18}F]KF-K_{222}-carbonate complex, the aqueous [^{18}F]fluoride (0.5–15 GBq) was added to a mixture of a solution of 11.2 mg (0.03 mmol) Kryptofix (K_{222}) in 1 mL CH_3CN and a solution of 1.8 mg (0.013 mmol) K_2CO_3 in 89 µL H_2O in a conical glass vial equipped with screw cap and rubber septum. This mixture was azeotropically dried by adding anhydrous MeCN in portions in the presence of a continuous stream of argon at 110 °C under stirring. The remaining [^{18}F]KF-K_{222}-carbonate complex was dissolved in the corresponding anhydrous reaction medium.

Preparation of 2-[^{18}F]fluoropropyl thiocyanate ([^{18}F]9). A solution of the corresponding 3-thiocyanatopropyl benzenesulfonate (**3** or **4**) in 500 µL of anhydrous CH_3CN or DMF was added to the dissolved anhydrous [^{18}F]KF-K_{222}-carbonate complex (0.5–5 GBq) and brought

to reaction at elevated temperature in the screw-cap-sealed vial. To optimise the reaction regarding the type and amount of precursor, reaction time and temperature, aliquots were withdrawn after 5, 10 and 15 min, diluted with 90 µL of CH_3CN and analysed by radio-TLC.

*Optional – Purification of [^{18}F]**9** by microdistillation.* The reaction vial containing crude [^{18}F]**9** was equipped with a screw cap containing a rubber septum and connected to a receiver tube with a Teflon tube. The receiver tube was equipped with a gas outlet, placed in a −60 °C isopropanol cooling bath and shielded with lead. A γ-detector was placed close to the receiver tube and the underground radiation was detected. The reaction vial was connected to an argon stream and heated to 80 °C in an oil bath. The radiation in the receiver tube was measured in time intervals and the underground was subtracted from the values. Within 10 min and a recovery of 92% (decay-corrected) of the [^{18}F]**9** was achieved (Figure 4 in main text).

Preparation of [^{18}F]9 by the optimised procedure. The residue containing the dried [^{18}F]fluoride (0.5–15 GBq) was dissolved in 400 µL of anhydrous CH_3CN. The mixture was transferred into a glass vial. A solution of 2.5–3.0 mg (9–11 µmol) of the tosylate precursor **3** or nosylate precursor **4** (8–10 µmol) in 250 µL anhydrous CH_3CN was added and reacted at 82 °C up to 15 min. Subsequently, the solution was cooled, diluted with 10 mL of water and passed through a Sep-Pak C18 cartridge (initially in 1 mL steps for checking the adsorption by activity measurements). The sorption was stopped after passing 7 mL of this solution through the cartridge to prevent the breakthrough of [^{18}F]**9**. Without washing, the C18 cartridge was blown-dry in a moderate argon stream for ~2 min and reacted with three portions of freshly prepared chlorine water (2.0, 1.0 and 0.5 mL; exposure time 2 min each) followed by drying with argon again. Finally, the C18 cartridge was eluted with dichloromethane (3 × 1 mL) using an intermediate Sep-Pak Dry (anhydrous sodium sulfate) cartridge for drying the eluates. The first fraction contained the major part of [^{18}F]**10** (~80%), the combined first two fractions about 95% of the activity. All steps were controlled by activity measurements. The solutions are available for further reactions. If necessary they can be concentrated by gentle evaporation of the solvent.

The use of two Sep-Pak C18 cartridges in sequence was advantageous for the solid-phase extraction of [^{18}F]**9**. Other types of C18 cartridges were tried but were not superior to Sep-Pak C18, resulting in either incomplete adsorption or quantitative adsorption accompanied by incomplete elution of [^{18}F]**9**.

Reproducible labelling yields of 45–55% (*n* = 9) and 75–85% (*n* = 12) for the reaction of **3** and **4**, respectively, with [^{18}F]F^- to [^{18}F]**9** could be achieved. [^{18}F]**10** was produced with isolated radiochemical yields of 40–45% (decay-corrected to start of synthesis) and a

radiochemical purity of 90–95% according to radio-TLC and analytical radio-HPLC within an overall synthesis time of 70 min.

Identification of [^{18}F]**9**: HPLC (conditions see above): t_R = 25.82 ± 0.12; TLC (petroleum ether/ethyl acetate 3:1): R_f = 0.61, (petroleum ether/ethyl acetate 1:1): R_f = 0.79 (nonradioactive reference compound was visualised under UV_{254}).

Identification of [^{18}F]**10**: HPLC (conditions see above): t_R = 37.26 ± 0.08; TLC (petroleum ether/ethyl acetate 3:1): R_f = 0.60 (nonradioactive reference compound was visualised by staining with a solution of sodium iodide in acetone [8]).

Representative procedures for the reaction of [^{18}F]10 with amines in the presence or absence of base

Aliphatic amines

To a solution of [^{18}F]**10** (125–1000 MBq) in CH_2Cl_2 (200 µL) 2–3 mg of the corresponding amine dissolved in 200 µL CH_2Cl_2 was added. After stirring for 3 min at room temperature, the mixture was analysed by radio-TLC (see Figure S1 for example) and analytical radio-HPLC, diluted with 600 µL of CH_3CN and 3 mL of water and subjected to semipreparative HPLC.

Aromatic amines (note the order of reagents!)

To a solution of 2–3 mg of the amine in CH_2Cl_2 (200 µL) 25–50% of the stoichiometric amount of a base (TEA, DMAP, KOTMS; see Table 2 in the main text) was added 200 µL [^{18}F]**10** (125–1000 MBq) dissolved in 200 µL each of CH_2Cl_2 (TEA and DMAP) or CH_2Cl_2/THF 3:1 (KOTMS). The resulting reaction mixtures were treated as described above.

Generally, the radiochemical yields for the 3-[^{18}F]fluoropropanesulfonamides could be determined in good agreement between radio-HPLC and radio-TLC within a deviation range of ±5%.

The purification of the 3-[^{18}F]fluoropropanesulfonamides was carried out via semipreparative HPLC (eluent: 42.5% CH_3CN/20 mM NH_4OAc; flow rate 1 mL/min) (see Figure S2 for example).

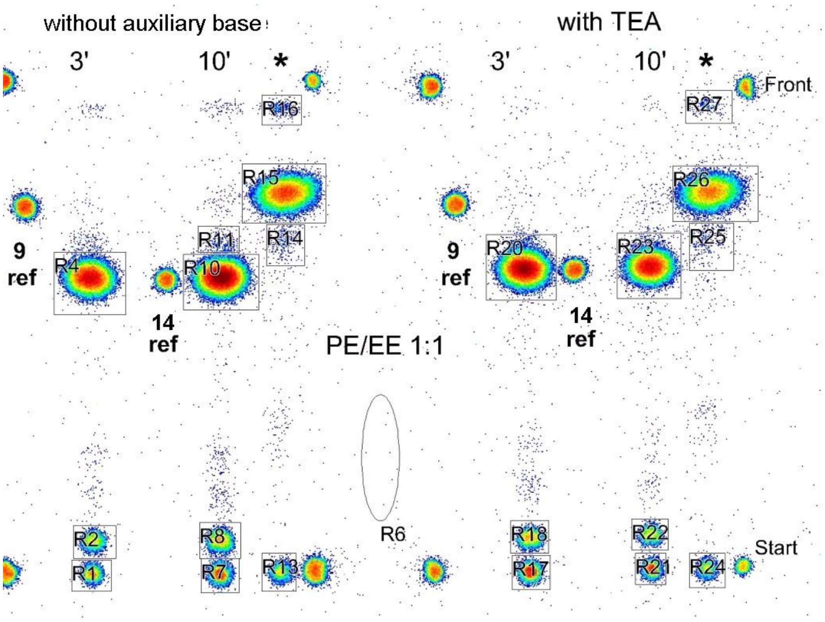

Figure S1: Radio-TLC analysis of the reaction of [^{18}F]**10** with benzylamine. The lane denoted by an asterisk represents [^{18}F]**10** as obtained after elution with dichloromethane and drying. Due to similar R_f values of compounds **9** and **10**, **9** was spotted for orientation as it is more easily visible under UV light.

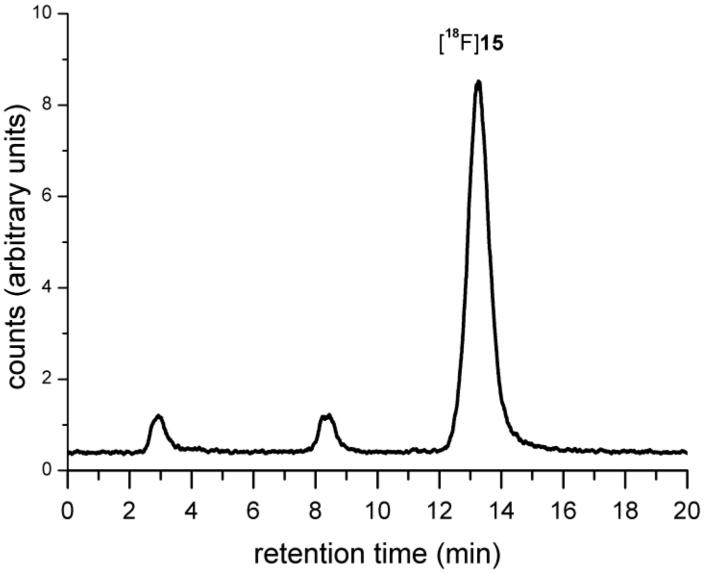

Figure S2: Semipreparative radio-HPLC for the radiosynthesis of [^{18}F]**15** from [^{18}F]**10** and cyclohexylamine. [^{18}F]**15** eluted at t_R = 13.2 min, i.e., sufficiently later than traces of residual [^{18}F]**10** (t_R = 11.2 min) and radioactive and UV-active by-products. [^{18}F]**15** could be prepared in an isolated radiochemical yield of 35–38% within a total synthesis time including the azeotropic drying of the [^{18}F]fluoride of 85–90 min (range of values from two independent experiments).

4. Stability studies for enzymatic degradation

The activity of the enzyme batch of carboxylesterase from porcine liver (pig liver esterase, PLE; Sigma E3019-3.5 kU, Lot# 028K7005V, 17 U/mg) was proven independently in a chromogenic assay using the standard substrate *para*-nitrophenyl butyrate. This was done by assaying the PLE activity in kinetic measurements at a temperature of 30 °C by detecting the product *p*-nitrophenol spectrophotometrically at a wavelength of 405 nm in a plate reader (BIOTEK Synergy 4) using transparent 96-well plates (final volume 200 µL). A 100 mM stock solution of the chromogenic substrate *para*-nitrophenyl butyrate (Sigma) was freshly prepared in DMSO; the final concentrations were 0.025, 0.05, 0.1, 0.2, 0.4, 0.8, and 1.0 mM. The assay medium was 10 mM potassium phosphate pH 7.4 containing 5% DMSO. The reactions were started by adding 10 µL each of a PLE solution (28 µg/mL) in 10 mM potassium phosphate pH 7.4 to each vial.

By fitting the parameters of the Michaelis–Menten equation (1)

$$v = \frac{V_{\max} \cdot [S]}{K_m + [S]} \quad (1)$$

to the experimental data using the programme GraphPad Prism 5 (GraphPad software) a Michaelis constant (K_m) of 743 µM could be determined (Figure S3). A value of K_m = 140 µM has been published without specification of the DMSO content [9].

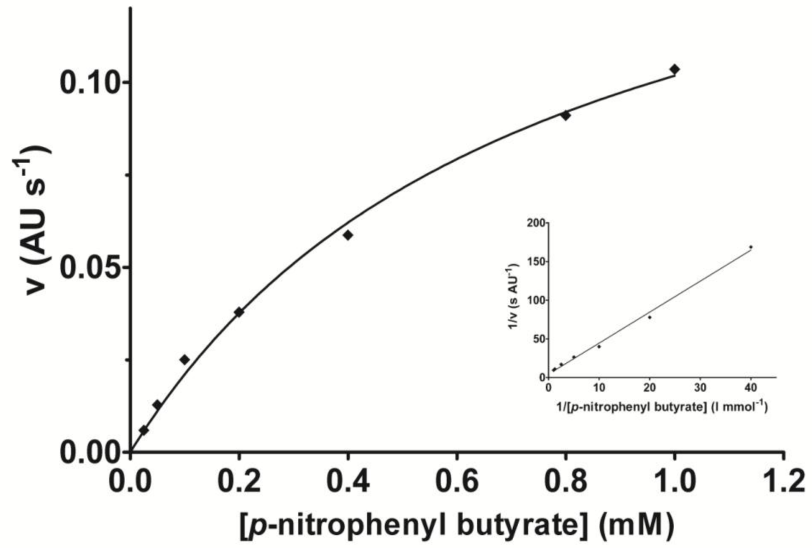

Figure S3: Determination of the Michaelis constant (K_m) towards *para*-nitrophenyl butyrate for the carboxylesterase from pig liver (pig liver esterase, PLE) used in the degradation experiments for compounds **17** and **19**. A K_m value of 743 ± 103 µM was calculated by nonlinear regression. The inset shows the Lineweaver–Burk plot to prove the quality of the data set. Conditions: 10 mM potassium phosphate pH 7.4, 5% DMSO, 30 °C, 1.4 µg/mL PLE.

Stock solutions of **17** and **19** (100 mM each) were prepared in DMSO. A solution of carboxylesterase from porcine liver (pig liver esterase, PLE); Sigma E3019-3.5 kU, Lot# 028K7005V, 17 U/mg) was prepared daily in 10 mM potassium phosphate pH 7.4 at a concentration of 28 mg/mL and kept on ice. Stock solutions of **17** and **19** were diluted to 1.05 mM with 10 mM potassium phosphate pH 7.4, a total DMSO content of 5.3% (v/v) was adjusted by adding the complementary volume of pure DMSO. 50 µL of the enzyme solution was added to each 950 µL of these solutions pretempered at 37 °C resulting in assay solutions composed of **17** or **19** at a concentration of 1 mM each, 10 mM potassium phosphate pH 7.4, 1.4 mg/mL PLE, and 5% DMSO (v/v). The solutions were kept at 37 °C. Aliquots of 20 µL were withdrawn every 30 min up to a total time of 180 min. An additional aliquot was withdrawn immediately after addition of the enzyme solution. Each aliquot was diluted with 20 µL of 2% CF_3COOH/CH_3CN. The resulting mixtures were centrifuged at 6000 rpm for 5 min. Each 30 µL of the supernatants (macroscopically, no sediment was visible) was injected into the HPLC apparatus under isocratic elution conditions ($H_2O/CH_3CN/CF_3COOH$ 1:1:0.001, v/v) at a flow rate of 1 mL/min. The compounds had the following retention times, 5.6 min (**17**) and 4.4 min (**19**).

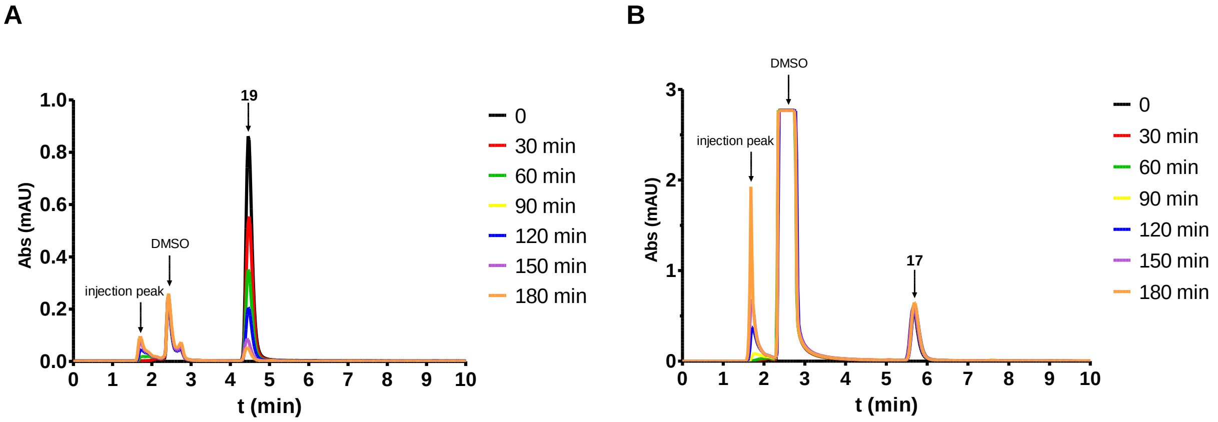

Figure S4: HPLC chromatograms for (**A**) the time course of the carboxylesterase-catalysed hydrolysis of **19** and (**B**) for sulfonamide **17** exposed to carboxylesterase.
Measurements were carried out in duplicate for each compound in independent experiments.

Chromatograms were extracted from the recorded DAD data for the wavelengths 240 nm (**19**; Figure S4A) and 223 nm (**17**; Figure S4B) and the peak areas were determined. Peak areas were converted to molar concentrations relative to the initial value and plotted against the time. Data evaluation was carried out with the program Graph Prism 5 by fitting the parameters c_0 and k_{obs} of the first-order rate law equation (2)

$$c = c_0 \cdot e^{-k_{obs} \cdot t} \quad (2)$$

to the experimental data.

5. X-ray structure determination

Crystallographic data were collected with a Bruker-Nonius Apex-X8 CCD-diffractometer with monochromatic Mo Kα radiation (λ = 0.71073 Å) and a CCD detector. Preliminary data of the unit cell dimensions was obtained from the reflex positions of 36 frames, measured in three different directions of the reciprocal space. After completion of the data measurement the reflex intensities were corrected for Lorentz, polarisation and absorption effects. The structure was solved by direct methods using SHELXS-97 and refined against F^2 on all data by full-matrix least-squares methods using SHELXL-97 version 2 [10,11]. All non-hydrogen atoms were refined anisotropically. Except for the hydrogen atom attached to the amide N-atom, H1C, all hydrogen atoms bonded to carbon atoms were placed on geometrically calculated positions and refined using riding models. H1C was refined isotropically. Crystallographic data has been deposited with the Cambridge Crystallographic Data Centre, CCDC-916522. It can be retrieved free of charge through deposit@ccdc.cam.ac.uk or http://www.ccdc.cam.ac.uk

6. References

1. Renard, P.-Y.; Schwebel, H.; Vayron, P.; Josien, L.; Valleix, A.; Mioskowski, C. *Chem.–Eur. J.* **2002,** *8,* 2910–2916. doi:10.1002/1521-3765(20020703)8:13<2910::AID-CHEM2910>3.0.CO;2-R
2. Li, Z.; Lang, L.; Ma, Y.; Kiesewetter, D. O. *J. Labelled Compd. Radiopharm.* **2008,** *51,* 23–27. doi:10.1002/jlcr.1466
3. Sukhai, R. S.; de Jong, R.; Verkruijsse, H. D.; Brandsma, L. *Recl. Trav. Chim. Pays-Bas* **1981,** *100,* 368–372. doi:10.1002/recl.19811001005
4. Pattison, F. L. M.; Millington, J. E. *Can. J. Chem.* **1956,** *34,* 757–768. doi:10.1139/v56-099
5. Howell, W. C.; Millington, J. E.; Pattison, F. L. M. *J. Am. Chem. Soc.* **1956,** *78,* 3843–3846. doi:10.1021/ja01596a078
6. Millington, J. E.; Brown, G. M.; Pattison, F. L. M. *J. Am. Chem. Soc.* **1956,** *78,* 3846–3847. doi:10.1021/ja01596a079
7. Bergmann, E. D.; Moses, P.; Neeman, M. *J. Sci. Food Agric.* **1957,** *8,* 400–404. doi:10.1002/jsfa.2740080706
8. Jardine, J. R.; Langler, R. F. *J. Chromatogr.* **1976,** *116,* 211–214. doi:10.1016/S0021-9673(00)83723-1
9. Landowski, C. P.; Lorenzi, P. L.; Song, X.; Amidon, G. L. *J. Pharmacol. Exp. Ther.* **2006,** *316,* 572–580. doi:10.1124/jpet.105.092726
10. Sheldrick, G. M., SHELXS/L-97, Programs for the Solution and Refinement of Crystal Structures, University of Göttingen, 1997.
11. Sheldrick, G. M. *Acta Crystallogr., Sect. A* **2008,** *A64,* 112–122. doi:10.1107/S0108767307043930
